# Supplementary material for: Predictors of falls and fractures leading to hospitalisation in 36 101 people with affective disorders: a large representative cohort study
Source: BMJ Open. 2022 Mar 11;12(3):e055070. doi: 10.1136/bmjopen-2021-055070 (PMC8919445; doi:10.1136/bmjopen-2021-055070)
Supplement: Supplementary data [file bmjopen-2021-055070supp001.pdf]

Supplementary material Table 1. Mean age at the time of fall/ fracture and mood disorder diagnosis among patients with Mood [affective] disorders

	Presence of falls		Presence of fractures	
Age	**No (n= 34,270)**	**Yes (n= 1,831)**	**No (n= 33,908)**	**Yes (n= 2,193)**
Mean age at diagnosis (SD)	43.4 (17.1)	62.4 (19.9)	43.3 (17.1)	60.6 (20.6)
Mean age at fall/ fracture/ (SD)		64.2 (20.8)		62.5 (22.8)
