# Supplementary material for: Predictors of falls and fractures leading to hospitalisation in 36 101 people with affective disorders: a large representative cohort study
Source: BMJ Open. 2022 Mar 11;12(3):e055070. doi: 10.1136/bmjopen-2021-055070 (PMC8919445; doi:10.1136/bmjopen-2021-055070)
Supplement: Author's manuscript [file bmjopen-2021-055070.draft_revisions.pdf]

BMJ Open is committed to open peer review. As part of this commitment we make the peer review history of every article we publish publicly available.

When an article is published we post the peer reviewers' comments and the authors' responses online. We also post the versions of the paper that were used during peer review. These are the versions that the peer review comments apply to.

The versions of the paper that follow are the versions that were submitted during the peer review process. They are not the versions of record or the final published versions. They should not be cited or distributed as the published version of this manuscript.

BMJ Open is an open access journal and the full, final, typeset and author-corrected version of record of the manuscript is available on our site with no access controls, subscription charges or pay-per-view fees (http://bmjopen.bmj.com).

If you have any questions on BMJ Open's open peer review process please email info.bmjopen@bmj.com

BMJ Open

Predictors of falls and fractures leading to hospitalization in 36,101 people with affective disorders: A large representative cohort study

Journal:	*BMJ Open*
Manuscript ID	bmjopen-2021-055070
Article Type:	Original research
Date Submitted by the Author:	05-Jul-2021
Complete List of Authors:	Ma, Ruimin; King's College London, Psychological Medicine<br>Perera, Gayan; King's College London, Psychological Medicine<br>Romano, Eugenia; King's College London, Psychological Medicine<br>Vancampfort, Davy; KU Leuven<br>Koyanagi, Ai; Parc Sanitari Sant Joan de Deu, Research and Development Unit<br>Stewart, Robert; King's College London, Institute of Psychiatry<br>Mueller, Christoph; King's College London, Department of Old Age Psychiatry<br>Stubbs, B; King's College London, Psychological Medicine
Keywords:	MENTAL HEALTH, Anxiety disorders < PSYCHIATRY, Adult psychiatry < PSYCHIATRY, Depression & mood disorders < PSYCHIATRY

SCHOLARONE™
Manuscripts

I, the Submitting Author has the right to grant and does grant on behalf of all authors of the Work (as defined in the below author licence), an exclusive licence and/or a non-exclusive licence for contributions from authors who are: i) UK Crown employees; ii) where BMJ has agreed a CC-BY licence shall apply, and/or iii) in accordance with the terms applicable for US Federal Government officers or employees acting as part of their official duties; on a worldwide, perpetual, irrevocable, royalty-free basis to BMJ Publishing Group Ltd ("BMJ") its licensees and where the relevant Journal is co-owned by BMJ to the co-owners of the Journal, to publish the Work in this journal and any other BMJ products and to exploit all rights, as set out in our licence.

The Submitting Author accepts and understands that any supply made under these terms is made by BMJ to the Submitting Author unless you are acting as an employee on behalf of your employer or a postgraduate student of an affiliated institution which is paying any applicable article publishing charge ("APC") for Open Access articles. Where the Submitting Author wishes to make the Work available on an Open Access basis (and intends to pay the relevant APC), the terms of reuse of such Open Access shall be governed by a Creative Commons licence – details of these licences and which Creative Commons licence will apply to this Work are set out in our licence referred to above.

Other than as permitted in any relevant BMJ Author's Self Archiving Policies, I confirm this Work has not been accepted for publication elsewhere, is not being considered for publication elsewhere and does not duplicate material already published. I confirm all authors consent to publication of this Work and authorise the granting of this licence.

For peer review only

Predictors of falls and fractures leading to hospitalization in 36,101 people with affective disorders: A large representative cohort study

Running title: Predictors of falls and fractures in people with affective disorders

Ruimin Ma[a^*], Gayan Perera[^a], Eugenia Romano[a], Davy Vancampfort[c, d], Ai Koyanagi[e, f], Robert Stewart[a, b], Christoph Mueller[^^a, b] Brendon Stubbs[^^a, b],

[a] Institute of Psychiatry, Psychology and Neuroscience (IoPPN), King's College London, London, United Kingdom

[b] South London and Maudsley NHS Foundation Trust, London, United Kingdom

[c] KU Leuven – University of Leuven, Department of Rehabilitation Sciences, Leuven, Belgium

[d] KU Leuven – University of Leuven, University Psychiatric Centre KU Leuven, Leuven, Kortenberg, Belgium

[e] Research and Development Unit, Parc Sanitari Sant Joan de Déu, Universitat de Barcelona, Fundació Sant Joan de Déu, Dr. Antoni Pujadas, 42 ,Sant Boi de Llobregat, Barcelona 08830, Spain

[f] ICREA, Pg. Lluis Companys 23, Barcelona, Spain

^ Joint first author

^^ Joint last author

- Corresponding author at: Psychological Medicine, Institute of Psychiatry, Psychology and Neuroscience (IoPPN), King's College London, De Crespigny Park, London, BOX SE5 8AF, United Kingdom.

  Email address: ruimin.1.ma@kcl.ac.uk

For peer review only - http://bmjopen.bmj.com/site/about/guidelines.xhtml

Abstract (257/300)

Objectives: To investigate predictors of falls and fractures leading to hospitalisation in people with affective disorders.

Design: Cohort study

Setting: the South London and Maudsley NHS Foundation Trust (SLaM) Biomedical Research Centre (BRC) Case Register

Participants: A large cohort of people with affective disorders (ICD10 codes F30-F34) diagnosed between January 2008 and March 2016 was assembled using data from the SLaM BRC Case Register

Primary and secondary outcome measures: Falls and fractures leading to hospitalisation were ascertained from linked national hospitalization data. Multivariable Cox proportional hazards analyses were administrated to identify predictors of first falls and fractures.

Results: Of 36,101 people with affective disorders (mean age 44.4 years, 60.2% female), 816 (incidence rate 9.91 per 1000 person years) and 1,117 (incidence rate 11.92 per 1000 person years) experienced either a fall or fracture respectively. In multivariable analyses, older age, analgesics use, increased physical illness burden, previous hospital admission due to certain co-morbid physical illnesses and increase in attendances to accident and emergency services following diagnosis were significant risk factors for both falls and fractures. Having a history of falls was a strong risk factor for recurrent falls, and a previous fracture was also associated with future fractures.

Conclusions: Over a mean 5 years' follow-up, approximately 8% of people with affective disorders were hospitalised with a fall or fracture. Several similar factors were found to predict risk of falls and fracture, for example, older age, comorbid physical disorders and

For peer review only

analgesic use. Routine screening for bone mineral density and falls prevention programmes should be considered for this clinical group.

Key words: osteoporosis, affective disorders, falls, fractures, hospital admission

Strength and limitation of the study:

- Predictors of falls and fractures leading to hospitalization in people with affective disorders were investigated with a large representative cohort
- Data of the study were derived directly from the electronic health record
- Falls and fractures leading to hospitalisation were ascertained from linked national hospitalization data
- We did not stratify according to different affective disorder subtypes and psychotropic medication categories
- We had no information on lifestyle factors and type of fractures recorded at admission

For peer review only

Word count: 3636/4000

Introduction

Falls and fractures are strongly related with increased morbidity, mortality, disability, and healthcare expenditure in the general population [1, 2], and they are a frequent issue in older adults [3]. Their causes are multifactorial, such as muscle weakness [4] and use of antiepileptic drugs [5]. Several government strategies have been implemented to prevent and assess falls in the general population [3, 6]. Fractures, for example, hip and spine fractures increase the risk of future falls and fractures [7]. Over 3 million people in the UK are at a high risk for fractures [8], and prevention has been acknowledged as a worldwide priority [9].

Despite some progress in the general population, there is poor evidence on risk factors for falls and fractures for patients with affective disorders. People with mental health problems like depression and schizophrenia report higher risks of falls and fractures compared to the general population [10, 11], and despite poor evidence in people with bipolar disorders, a few studies suggest this population may also be at increased risk [12, 13].

Several studies have investigated the association between bone loss and depression, but there is a lack of large-scale cohort studies focussing on falls and fractures and related predictors. Another major limitation is that the majority of studies have relied on self-reported affective symptoms rather than clinical diagnosis [14, 15].

Given these limitations and the increased concerns about the adverse events of falls and fractures, this representative cohort study investigates predictors of falls and fractures leading to hospitalisation among people with clinically diagnosed affective disorders.

Methods

For peer review only

A retrospective observational study was carried out using data from the South London and Maudsley NHS Foundation Trust (SLaM) Biomedical Research Centre (BRC) Case Register. SLaM is one of the largest mental health and dementia care providers in Europe, serving a geographic catchment of four South London Boroughs (including Lambeth, Lewisham, Southwark, and Croydon) with a population in excess of 1.3 million. Data for this study were retrieved using the Clinical Record Interactive Search (CRIS) platform, which enables a de-identified version of SLaM's electronic health record to be accessible for research projects within a robust and patient-led government framework [16]. The SLaM BRC Case Register has been described in detail elsewhere [17] and has supported a wide range of studies [18, 19], including longitudinal cohort studies investigating falls and fractures in other populations [20, 21]. Data are currently archived in CRIS on more than 450,000 cases with a wide variety of mental disorders. CRIS has full approval for secondary analysis (Oxford Research Ethnic Committee C, reference 18/SC/0372). Data from source structured fields have been extensively supplemented through natural language process (NLP) applications using Generalised Architecture for Text Engineering software, applying information extraction techniques to derive structured information from the extensive text fields held in the mental health record [17].

For peer review only

Participants, study period and additional data sources

All SLaM patients with mood [affective] disorders (ICD10 codes F30-F34), diagnosed between 1st of January 2008 and 31st March 2016, were included. SLaM patient records have been linked with national Hospital Episode Statistics (HES) which are compiled from all NHS Trusts in England (both acute and mental health services), including statistical abstracts of records of all inpatient episodes, as well as outpatient and emergency care [17]. In addition,

For peer review only - http://bmjopen.bmj.com/site/about/guidelines.xhtml

CRIS data have been linked to the Office for National Statistics (ONS) mortality records over the same period.

Patient and public involvement (PPI)

The entire research program was developed after extensive patient, public and carer input, who helped develop the study, and contributed to the funding applicable. They co-developed the research questions, outcomes and contributed to factors included in the models. Moreover, they reinforced this research as a neglected topic of utmost importance to them. The PPI group designed the study with the senior researcher of the paper (Dr BS), they were involved in the conception of the idea, the planning of the study during bimonthly meetings to discuss study design, hypotheses, outcomes and what matters to patients of interest. They were also involved in helping interpret the data outputs and publication. Results will be summarised and made available to the PPI group through local newsletters and inform clinical services and local co-developed Recovery College Courses.

Co-primary outcome: falls and any fractures

The co-primary outcomes were hospital admissions relating to a fall or any fracture extracted from linked HES data and all discharge diagnoses (primary or any secondary diagnosis codes) recorded between January 2008 to March 2016, and based on the following ICD 10 codes: i) falls (W00-W19); ii) fractures (M80-M84, M907, S02, S12, S32, S42, S52, S62, S72, S82, S92, S22, T02, T08, T10, T12X, T902, T911, T912, T921). In addition, linked mortality records from the ONS linkage were examined for any instance of fall or fracture ICD codes in

For peer review only

any cause of death field on the death certificate to identify the date of death attributed to a fall or fracture.

Measurements

Several additional measurements were obtained from CRIS. All independent variables (covariates) were defined according to the value closest to the date of the first recorded mood [affective] disorder diagnosis. Demographic covariates comprised: age at diagnosis, gender, and ethnicity [multiple codes categorised into 1) White, consists of White British, White Irish and other White; and 2) Non-White]. Index of Multiple Deprivation (IMD 2015) for the neighbourhood of residence (i.e. Lower Super Output Area, which consists of approximately 650 households) at the time of diagnosis was divided into quintiles according to deprivation scores with equal size allocated to each group. The IMD has previously been used in CRIS [22] and combines Census-derived data at area level across several domains including income, employment, health, education, barriers to housing and services, living environment, and crime [23]. Information on cohabiting status (Cohabiting: married/civil partner, married, cohabiting; Non-cohabiting: single, divorced, civil partnership dissolved, widowed, separated) was also ascertained at the index diagnosis.

Illness burden

The Health of the Nation Outcome Scales (HoNOS) [24] are routinely administered measures of illness burden in UK mental health services and are recorded in structured fields on the electronic health record. Individual HoNOS item scores (agitated behaviour, self-injury, problem drinking & drugs, cognitive problems, physical illness, hallucinations, depressed mood, relationship problems, daily living problems, living conditions problems, occupational

For peer review only - http://bmjopen.bmj.com/site/about/guidelines.xhtml

problems) and dates were obtained within 6 months before or after the date of the index diagnosis, and the closest scores in time to this date were included in analyses. Each HoNOS item is rated on a Likert Scale, ranging from 0 (i.e. no problem) to 4 (i.e. severe or very severe problem). A detailed glossary for HoNOS is reported elsewhere [25]. Scores 2 or over in the individual HoNOS scales were classified as having a problem on each item, generating binary covariates.

Mental disorder comorbidity

Diagnoses of F00-F03 (dementia); F20-F29 (schizophrenia spectrum disorder); F40-F48 (neurotic, stress-related and somatoform disorders); F50 (eating disorders); F60-F69 (disorders of adult personality and behaviour) were ascertained within one year before or after the index diagnosis of mood [affective] disorder.

Medication

Medications received were extracted from structured medication fields in the record, supplemented by an NLP application applied to text fields ascertaining mentions of current medication [17]. Presence or not of the following medication groups was ascertained on the basis of information within six months before or after the index diagnosis: anticholinergics, antihypertensives, antidepressants, antipsychotics, anxiolytics and hypnotics, and analgesics. The total number of medications prescribed for any condition was calculated for each participant and used as a continuous variable.

For peer review only - http://bmjopen.bmj.com/site/about/guidelines.xhtml

Physical comorbidity

Information on physical comorbidities was ascertained utilising the data linkage between CRIS and national HES records. Information on all ICD-10 diagnoses at discharge (primary or any secondary diagnosis codes) was ascertained from any hospitalisations within 6 months before or after the index diagnosis date and the following binary variables generated: i) Ischaemic heart disease (IHD; I20, I21, I22, including coronary heart disease (CHD; I25); ii) Arrhythmia (I44-I49, including atrial fibrillation (AF; I48); iii) Heart failure (I50); iv) Diabetes (E08, E09, E10, E11, E12, E13; v) Hypotension (I95-99); vi) Hypercholesterolemia (E78); vii) Hypertension (I10-15); viii) Urinary tract infections (UTI) (N39); ix) Osteoporosis (M80-85); x) Visual disturbance and blindness (H53-54); xi) Hearing loss (H90-95); xii) Syncope or collapse (R50-R69); xiii) Parkinson's disease (G20). Furthermore, occurrence of falls and/or fractures before the index diagnosis was also collected. Finally, the number of attendances to accident and emergency services (A&E) following the index diagnosis was also recorded.

Statistical analysis

The study sample was described initially in terms of demographic and clinical variables, followed by unadjusted Cox proportional hazard models to predict the first fall and first fracture separately after the index diagnosis of mood [affective] disorder. The predictor variables at baseline used in the first univariate models included sociodemographic information (year of index diagnosis, mean age, gender, ethnicity, marital status), medications (antipsychotics, anxiolytics & hypnotics, antidepressants, analgesics, anticholinergics, antihypertensives), comorbid psychiatric diagnosis (F20-29 schizophrenia spectrum disorder, F40-48 neurotic/stress disorders, F00-F03 dementia, F50 eating disorders, F60 disorders of adult personality and behaviour), HONOS scores (mean total and each individual item) and physical

For peer review only - http://bmjopen.bmj.com/site/about/guidelines.xhtml

health comorbidities (as indicated above). Factors that yielded a p-value < 0.10 in the univariate model for fall or fracture outcome were subsequently entered into the multivariable model. A final multivariable Cox proportional hazards model, using stepwise backward elimination technique where those variables not significant (p-value > 0.05) were eliminated, with hazard ratios and 95% confidence intervals (CI) displayed. Having checked a correlation matrix of coefficients in the Cox model, total number of medications received was substantially collinear with individual types of medication received; therefore, this total number was removed as a covariate. All analyses were conducted utilising STATA, version 13.

Results

The sample comprised 36,101 people with a diagnosis of mood [affective] disorder (F30* - F34*) (mean age at first diagnosis 44.4, SD: 17.8, 60.2% female). Over a mean 5 years' follow-up, 2,948 patients had a fall and/or a fracture recorded: 816 only a fall, 1,117 only a fracture, and 1,015 both; 1,831 with any fall and 2,193 with any fracture. The incidence rate of falls was 9.91 per 1,000 person years and that for fractures 11.92 per 1,000 person years. Table 1 summarises characteristics of those who had a recorded fall or fracture compared to those who did not.

[Table 1]

Length of hospital stay

The mean length of hospitalisation following a fall (n = 1,831) was 7.9 days (range 0-374), for a total of 20,767 full days in hospital. The mean length of stay in hospital following a fracture

For peer review only - http://bmjopen.bmj.com/site/about/guidelines.xhtml

For peer review only

(n=2,193) was 13.2 days (range 0-374), for a total of 40,548 days. This equates to 18.51 years of inpatient hospital stay for 1,000-person years of follow-up due to a fall and 36.15 years of inpatient hospital stay for 1,000-person years of follow-up due to a fracture. For the 1,831 patients reporting a fall, the mean hospital admissions due to a fall was 1.5 (range 1- 15); for the 2,193 patients reporting a fracture, the mean number of hospital admissions due to a fracture was 2.3 (range 1-42).

Factors associated with falls and fractures

Cox proportional hazard models analysing unadjusted predictors of falls and fractures (95% CI) are reported in Table 2.

[Table 2]

Multivariable predictors of falls

Multivariable models of factors associated with first fall hospital admission are presented in Table 3. In Model 2, older age was strongly associated with higher risk, and Non-European ethnicity with lower risk, but neighbourhood deprivation reported no association. Analgesics had a significant association (with increased risk), and of co-morbid psychiatric conditions, only the ICD-10 F40-F48 group had a significant association (with reduced risk). Of the HoNOS items, cognitive problems and physical illness were associated with higher risk, and depressed mood problems with lower risk. Higher risk of falls was associated with several hospitalisation discharge diagnoses, namely heart failure, diabetes, hypotension, UTI, osteoporosis, and syncope or collapse. Hospitalised fall was associated with having a previous

For peer review only - http://bmjopen.bmj.com/site/about/guidelines.xhtml

For peer review only

history of falls reported before the index diagnosis and with increased A&E attendance after the index diagnosis.

[Table 3]

Multivariable predictors of fractures

Table 4 presents multivariable models of factors associated with first fracture hospitalisation. Fracture risk was increased in older patients and women and was decreased in those of Non-European. Fracture risk was lower in patients receiving antihypertensives and higher in those receiving analgesics. It was also lower in those with neurotic, stress-related and somatoform disorders (i.e. F40-F48), as well as bipolar affective disorder and manic episodes (i.e. F30-F31). An elevated fracture risk was associated with physical illness on the HoNOS. Fracture risk was independently predicted by previous hospitalisations for heart failure, diabetes, UTI, osteoporosis, hearing loss and preceding fracture-related (but not fall-related) hospitalisations. Fracture risk was also associated with higher levels of post-diagnostic A&E attendance.

[Table 4]

For peer review only

Discussion

To our knowledge, the current study is the first using representative data to investigate the predictors of hospitalised falls and fractures in people with clinically diagnosed affective disorders. Our data suggest that out of 36,101 people with affective disorders, 816 (i.e. 2.26%) and 1,117 (i.e. 3.09%) of patients experienced a fall and fracture, respectively. Length of hospital stay was considerable, equating to 18.51 and 36.15 years of inpatient hospital stay for 1,000-person years of follow-up due to a fall and a fracture, respectively. The key factors increasing the risk of a hospitalised fall were older age, analgesic use, increased illness burden due to cognitive problems and physical illness, a history of general hospital admission due to co-morbid physical illnesses (in particular syncope or collapse), increase in one attendance to A & E following affective disorder diagnosis, and falls before the diagnosis. Similar risk factors for fractures were noted.

Approximately 8.2% of our sample experienced a hospital admission due to either a fall or fracture. Studies on healthy populations have suggested several mechanisms which may explain these elevated risks in our sample, such as vitamin D deficiency [26, 27] and use of antidepressants [28]. Several studies also proposed a negative effect of leptin on bone mass and bone formation through a hypothalamic relay [29]. Some lifestyle factors seem to also be associated with a poor bone health, including physical inactivity [30] and alcohol consumption [31]. Previous studies have hypothesised other risk factors contributing to falls and fractures in people with depressive disorders [32], but no large-scale study has yet focused on patients with clinically diagnosed affective disorders. Our data advance the current literature by providing detailed evidence on how a multitude of demographic, medical, and psychological factors can differently affect risks of falls and fractures in people with affective disorder.

For peer review only

The association between older age and incidence of fall may be due to age-related physical conditions, as well as physiologic (e.g. loss of BMD) [33] and pathologic changes (e.g. decreased cardiovascular functions) [34]. Female gender is also a well-established risk factor for osteoporosis [35], consistent with our finding. Surprisingly, we found no relationship between deprivation and falls and fractures, although previous evidence has associated deprivation with fractures across different age groups [36, 37]. We might suppose that while deprivation is associated with poor nutrition and physical inactivity, people with affective disorders have a poorer lifestyle, compared to the general population [38], regardless of their financial status or living condition. Finally, the protective role of a non-European ethnic background is comparable with previous evidence supporting a higher fracture rate in Caucasian women compared to women from other ethnic groups [39], and a lower fall rate in older immigrants than those with an English-speaking background [40].

Our results provide new evidence on how hospitalisation due to falls or fractures can be an important burden for the NHS resources and for people with affective disorders, who are already at risk for a prolonged hospitalisation [41]. Given the long hospital stay among our sample, its burden on NHS costs in unsurprising. The total cost of fracture is estimated to reach £4.4 billion per year in the UK alone [8]. Several items from the HoNOS were also associated with increased hazard of hospitalisation due to falls or fractures (e.g. cognitive problems). Evidence on the role of these factors on hospital stay among this population is warranted for future research.

For peer review only - http://bmjopen.bmj.com/site/about/guidelines.xhtml

Our study reports stress-related and somatoform disorders to be associated with a decreased risk of falls and fractures, which could be due to increased fear avoidance and reduced activities due to stress-related disorders, leading to fewer falls [42]. We also found the presence of bipolar affective disorder and manic episodes to be protective factors against increased fracture risk. Although there has been little evidence investigating the role of bipolar affective disorder or its characteristics (including manic symptoms) in the risk of fracture, preliminary evidence has suggested an association between the use of lithium (i.e. a primary treatment for bipolar disorder) and decreased fracture risk [43], the preservation and enhancement of bone mass [44].

Surprisingly, neither antidepressants nor antipsychotics were associated with risks of falls or fracture, despite documented associations between antipsychotic medications and reduced bone metabolism [45] and BMD [46]. Evidence on the association between antidepressants and BMD is ambiguous: while some authors reported that some antidepressants, especially SSRIs, have a negative impact on bone strength [47, 48], other findings have been null [49]. However, we did not attempt to account for specific types of antidepressants in the analysis, and previous studies indicate that tricyclic antidepressants have no adverse effect on bone health [28]. On the other hand, analgesics use was associated with increased risks for falls and fractures, consistently with previous findings [50], and possibly mediated by central nervous system effects, such as dizziness [51]. Antihypertensive drugs use was associated with a decreased fracture risk, confirming previous results [52], potentially due to their improving effect on calcium absorption [53].

Finally, extensive evidence has indicated several physical conditions as risk factors for falls or fractures in the general population [54, 55], including UTI [56] and diabetes [57]. It is known

For peer review only

that mental health disorders (e.g. depression) increase the chance of major physical co-morbidities [58]. However, few studies have established the impact of comorbid physical conditions on risks of falls and fractures among people with affective disorders. Our study indicates heart failure, diabetes, UTI and osteoporosis to be associated with hospitalisation due to falls or fractures. Hypotension and syncope or collapse were additionally associated with an increased risk of falls, and hearing loss was associated with an elevated fracture risk. Unsurprisingly, osteoporosis was strongly associated with falls and fractures, as it has been acknowledged as the most important yet potentially treatable factor for falls [54] and fractures [59] due to its effect on BMD. Moreover, osteoporosis tends to occur simultaneously with heart failure [60] due to shared risk factors such as diabetes. Unsurprisingly, syncope or collapse was also strongly associated with an increased risk of falls, as syncope is frequently mistaken for falls, to the point that the European Society of Cardiology has emphasised the need to explore syncope as an independent factor leading to falls [61]. Diabetes was another risk factor for both falls and fracture, confirming the association found in the general population [62]. Hearing loss was the only factor associated with an elevated risk of fracture in our study, an association previously confirmed in the general population [63]. This finding may be explained by the damaging impact of hearing loss on orientation skills and physical activity [64]. Finally, our study confirmed the association between history of falls and increased risk of future falls, previously reported in the general population [34, 65] and in people with depression [66]. Moreover, our data confirmed how a prior fracture represents a 50-100% higher risk for experiencing future fracture, as reported in the general population [67].

The main strengths of our study are: i) analyses controlling for various confounders; and ii) a large sample including people with clinically diagnosed affective disorders. However, there are important limitations. Firstly, we did not stratify according to different affective disorder

For peer review only

subtypes which could report different risk factors; similarly, we analysed psychotropic medication categories but did not investigate specific agents or sub-categories. Second, we had no information on the types of fractures reported at admission and on factors (e.g. balance) that are influenced by neuropathological lesions in people with depression [68]. Third, lifestyle factors, such as alcohol consumption [31, 38], could not be explored. Fourth, our study only ascertained information on physical co-morbidities from hospitalisation records and could have overlooked patients with mild osteoporosis. Additionally, physical co-morbidities were ascertained within six months of diagnosis, and patients who were hospitalised for a fall or fracture within these six months would be more likely to have their osteoporosis ascertained. Finally, since medication records were ascertained within one year of diagnosis, we cannot assess whether medications were prescribed as a result or as a cause of falls for those who had a fall or fracture within six months of their diagnosis.

Conclusion

Our study reports that over a mean 5 years' follow-up, approximately 8% of a large cohort with affective disorders was hospitalised due to either a fall or fracture. Factors such as older age, certain medications and having a history of fall or fracture are significant predictors, broadly comparable to risk factors established in the general population. Heavy hospital burden following a fall or fracture was also described.

Our current study provides important implications for future research and clinical practice. BMD checks and osteoporosis screenings should be a routine for people with affective disorders, especially if older and with established co-morbid chronic illnesses. However, evidence suggests a lack of osteoporosis screening and fracture prevention for people with

For peer review only - http://bmjopen.bmj.com/site/about/guidelines.xhtml

mental health problems [69]. Fall assessments (e.g. the Fracture Risk Assessment Tool) should be considered for people with affective disorders, since approximately 70% of low-energy fractures are due to falls [70]. However, there is a lack of fall prevention programmes for this population, despite research indicating an average 14% reduction in falls risk in the general population [71]. Fall/fracture prevention programmes should therefore be offered; for example, the combination of cognitive behavioural therapy and exercise for improving depressive symptoms [72] and self-efficacy [73].

Acknowledgments

Author's contributions

BS acquired funding for the study. GP conducted the analysis with support from all co-authors. RM drafted introduction and discussion sections, ER drafted results section and all authors (BS, GP, DV, AK, RS, CM) provided critical revisions and approved the final version. The PPI group helped conceptualize and co-develop the study, as well as contributed to the funding applications.

Financial support

BS is supported by a Clinical Lectureship (ICA-CL-2017-03-001) jointly funded by Health Education England (HEE) and the National Institute for Health Research (NIHR). BS and RS are part funded by the NIHR Biomedical Research Centre at South London and Maudsley NHS Foundation Trust. RM is part funded by the HDR-UK London award to King's College London. The views expressed are those of the author(s) and not necessarily those of the (partner organisation), the NHS, the NIHR or the Department of Health and Social Care.

For peer review only

Ethics approval

This study has full approval for secondary analysis of CRIS (Oxford Research Ethnic Committee C, reference 18/SC/0372).

Conflicts of interest

All authors declare no conflicts of interest.

Data availability statement

Data are available on reasonable request. For questions regarding the study, please contact RM at ruimin.1.ma@kcl.ac.uk

For peer review only

References

[1] Hernlund E, Svedbom A, Ivergård M, Compston J, Cooper C, Stenmark J, McCloskey EV, Jönsson B, Kanis JA. Osteoporosis in the European Union: Medical management, epidemiology and economic burden: A report prepared in collaboration with the International Osteoporosis Foundation (IOF) and the European Federation of Pharmaceutical Industry Associations (EFPIA). *Archives of Osteoporosis*. 2013 https://doi.org/10.1007/s11657-013-0136-1

[2] Sattui SE, Saag KG. Fracture mortality: Associations with epidemiology and osteoporosis treatment. *Nature Reviews Endocrinology*, 2014; *10*, 592–602.

[3] Panel on Prevention of Falls in Older Persons, American Geriatric Society and British Geriatric Society. Summary of the updated American Geiratric Society/British Geiratric Society clincial practice guideline for prevention of falls in older persons. Journal of American Geiratric Society, 2011; 59(1): 148-157

[4] Dionyssiotis Y. Analysing the problem of falls among older people. International Journal of General Medicine, 2012; 5: 805-813.

[5] Deandrea S, Lucenteforte E, Bravi F, Foschi R, La Vecchia C, Negri E. Risk factors for falls in community-dwelling older people: A systematic review and meta-analysis. In *Epidemiology* 2010: pp. 658–668. https://doi.org/10.1097/EDE.0b013e3181e89905

[6] NICE. Falls in older people: assessing risk and prevention. 2013. Available at https://www.nice.org.uk/guidance/cg161. Accessed 13 March 2020

[7] Sanguineti VA, Wild JR, Joseph B, Fain MJ. Management of common fractures in older adults. In Michel, J-P., Beattie, B. L., Martin, F. C., & Walston, J (eds). Oxford Textbookof Geriatric Medictine (3ed).2017. Oxford University Press. DOI:

For peer review only - http://bmjopen.bmj.com/site/about/guidelines.xhtml

10.1093/med/9780198701590.003.0070

[8] Public Health England. Falls: applying all our health. 2020. Available at https://www.gov.uk/government/publications/falls-applying-all-our-health/falls-applying-all-our-health. Accessed 13 March 2020

[9] Johnell O, Kanis JA. An estimate of the worldwide prevalence and disability associated with osteoporotic fractures. Osteoporos. Int. 2006; 17 (12): 1726–1733

[10] Correll CU, Detraux J, De Lepeleire J, De Hert M. Effects of antipsychotics, antidepressants and mood stabilizers on risk for physical diseases in people with schizophrenia, depression and bipolar disorder. World Psychiatry, 2015; 14, 119–136. https://doi.org/10.1002/wps.20204

[11] Shi TT, Min M, Zhang Y, Sun CY, Liang MM, Sun YH. Depression and risk of hip fracture: a systematic review and meta-analysis of cohort studies. In Osteoporosis International, 2019; 30(6): 1157–1165. https://doi.org/10.1007/s00198-019-04951-6

[12] Chandrasekaran V, Brennan-Olsen SL, Stuart AL, Pasco JA, Berk M, Hodge JM, Williams LJ. Bipolar disorder and bone health: A systematic review. Journal of Affective Disorders, 2019; 249: 262–269. https://doi.org/10.1016/j.jad.2019.02.013

[13] Hong-Jhe C, Chin-Yuan, K, Ming-Shium T, Fu-Wei W, Ru-Yih C, Kuang-Chieh H, Hsiang-Ju P, Ming-Yueh C, Pan-Ming C, Chih-Chuan P. The incidence and risk of osteoporosis in patients with anxiety disorder: A Population-based retrospective cohort study. *Medicine*, 2016; *95*(38): e4912. https://doi.org/10.1097/MD.0000000000004912

[14] Wu Q, Liu B, Tonmoy S. Depression and risk of fracture and bone loss: an updated meta-analysis of prospective studies. *Osteoporosis International*, 2018; *29*: 1303–1312. https://doi.org/10.1007/s00198-018-4420-1

For peer review only

[15] Stubbs B, Stubbs J, Gnanaraj SD, Soundy A. Falls in older adults with major depressive disorder (MDD): a systematic review and exploratory meta-analysis of prospective studies. Int Psychogeriatr, 2016; 28(1): 23-29. doi: 10.1017/S104161021500126X.

[16] Fernandes AC, Cloete D, Broadbent MTM, Hayes RD, Chang CK., Jackson RG, et al. Development and evaluation of a de-identification procedure for a case register sourced from mental health electronic records. *BMC Medical Informatics and Decision Making*, 2013; *13*: 71. https://doi.org/10.1186/1472-6947-13-71

[17] Perera G, Broadbent M, Callard F, Chang CK, Downs J, Dutta R, et al. Cohort profile of the South London and Maudsley NHS Foundation Trust Biomedical Research Centre (SLaM BRC) Case Register: Current status and recent enhancement of an Electronic Mental Health Record-derived data resource. *BMJ Open*, 2016; *6*(3): e008721. https://doi.org/10.1136/bmjopen-2015-008721

[18] Sultana J, Chang CK, Hayes RD, Broadbent M, Stewart R, Corbett A, Ballard C. Associations between risk of mortality and atypical antipsychotic use in vascular dementia: A clinical cohort study. *International Journal of Geriatric Psychiatry*, 2014; *29*(12): 1249–1254. https://doi.org/10.1002/gps.4101

[19] Ward G, Perera G, Stewart R. Predictors of mortality for people aged over 65 years receiving mental health care for delirium in a South London Mental Health Trust, UK: A retrospective survival analysis. *International Journal of Geriatric Psychiatry*, 2015; *30*(6): 639–646. https://doi.org/10.1002/gps.4195

[20] Mueller C, Perera G, Hayes RD, Shetty H, Stewart R. Associations of acetylcholinesterase inhibitor treatment with reduced mortality in Alzheimer's disease: A retrospective survival analysis. *Age and Ageing*, 2018; *47*: 88–94. https://doi.org/10.1093/ageing/afx098

For peer review only - http://bmjopen.bmj.com/site/about/guidelines.xhtml

[21] Stubbs B, Mueller C, Gaughran F, Lally J, Vancampfort D, Lamb SE, et al. Predictors of falls and fractures leading to hospitalisation in people with schizophrenia spectrum disorder: a large representative cohort study. Schizophrenia Research, 2018; 201: 70-78

[22] Das-Munshi J, Chang C-K, Dutta R et al. Ethnicity and excess mortality in severe mental illness: a cohort study. The Lancet 2017; 4(5): 389-399. Doi: https://doi.org/10.1016/S2215-0366(17)30097-4

[23] Richardson EA, Mitchell RJ, Shortt NK et al. Evidence-based selection of environmental factors and datasets for measuring multiple environmental deprivation in epidemiological research. Environ Health, 2009; 8: S18. DOI: https://doi.org/10.1186/1476-069X-8-S1-S18

[24] Wing JK, Beevor AS, Curtis RH, Park SGB, Hadden J, Burns, A. Health of the Nation Outcomes Scales (HoNOS): Research and development. The British Journal of Psychaitry, 1998; 172(1):11-18. DOI: https://doi.org/10.1192/bjp.172.1.11

[25] Burns A, Beevor A, Lelliott P, Wing J, Blakey A, Orrell M, et al. Health of the Nation Outcome Scales for Elderly People (HoNOS 65+): Glossary for HoNOS 65+ score sheet. Cambridge University Press, 1999; 174(5): 435-438.
DOI: https://doi.org/10.1192/bjp.174.5.435

[26] Kahl KG, Greggersen W, Rudolf S, Stoeckelhuber BM, Bergmann-Koester CU, Dibbelt L, Schweiger U. Bone mineral density, bone turnover, and osteoprotegerin in depressed women with and without borderline personality disorder. *Psychosomatic Medicine*, 2006; *68*: 669–674. https://doi.org/10.1097/01.psy.0000237858.76880.3d

[27] Kahl KG, Rudolf S, Dibbelt L, Stoeckelhuber BM, Gehl HB, Hohagen F, Schweiger U. Decreased osteoprotegerin and increased bone turnover in young female patients with major depressive disorder and a lifetime history of anorexia nervosa. *Osteoporosis*

For peer review only

For peer review only - http://bmjopen.bmj.com/site/about/guidelines.xhtml

International, 2005; *16*: 424–429. https://doi.org/10.1007/s00198-004-1711-5

[28] Diem SJ, Blackwell TL, Stone KL, Yaffe K, Haney EM, Bliziotes MM, Ensrud, KE. Use of antidepressants and rates of hip bone loss in older women: The study of osteoporotic fractures. *Archives of Internal Medicine*, 2007; *167*: 1240–1245. https://doi.org/10.1001/archinte.167.12.1240

[29] Trivedi R, Goswami R, Chattopadhyay N. Invesgitational anabolic therapies for osteoporosis. Expert Opin Investig Drugs, 2010; 19(8): 995-1005

[30] Wong SYS, Lau EMC, Lynn H, Leung PC, Woo J, Cummings SR, Orwoll E. Depression and bone mineral density: Is there a relationship in elderly Asian men? Results from Mr. Os (Hong Kong). Osteoporosis International, 2005; 16: 610–615. https://doi.org/10.1007/s00198-004-1730-2

[31] Mussolino ME. Depression and hip fracture risk: The NHANES I epidemiologic follow-up study. Public Health Reports, 2005; 120: 71–75. https://doi.org/10.1177/003335490512000112

[32] Cizza G. Major depressive disorder is a risk factor for low bone mass, central obesity, and other medical conditions. Dialogues in Clinical Neuroscience, 2011; 13(1): 73–87.

[33] Wu Q, Magnus JH, Liu J, Bencaz AF, Hentz JG. (2009). Depression and low bone mineral density: A meta-analysis of epidemiologic studies. Osteoporosis International, 2009; 20(8): 1309–1320. https://doi.org/10.1007/s00198-009-0918-x

[34] Ambrose AF, Paul G, Hausdorff JM. (2013). Risk factors for falls among older adults: A review of the literature. Maturitas, 2013; 75(1): 51–61. https://doi.org/10.1016/j.maturitas.2013.02.009

[35] Williams J, Kool B, Robinson E,Ameratunga S. Longer term health of young and

For peer review only

middle-aged adults following unintentional falls at home resulting in hospitalisation. *Injury*, 2012; *43*(1): 103–108. https://doi.org/10.1016/j.injury.2011.03.050

[36] Court-Brown CM, Aitken SA, Duckworth AD, Clement ND, McQueen MM. The relationship between social deprivation and the incidence of adult fractures. *Journal of Bone and Joint Surgery - Series A*, 2013; *95*(6): e321–e327. https://doi.org/10.2106/JBJS.K.00631

[37] Menon MRG, Walker JL, Court-Brown CM. The epidemiology of fractures in adolescents with reference to social deprivation. *Journal of Bone and Joint Surgery - Series B*, 2008; *90*(11): 1482–1486. https://doi.org/10.1302/0301-620X.90B11.21163

[38] Eskandari F, Martinez PE, Torvik S, Phillips TM, Sternberg EM, Mistry S, et al. Low bone mass in premenopausal women with depression. *Archives of Internal Medicine*, 2007; *167*: 2329–2336. https://doi.org/10.1001/archinte.167.21.2329

[39] Cauley JA, Chalhoub D, Kassem AM, Fuleihan GEH. Geographic and ethnic disparities in osteoporotic fractures. *Nature Reviews Endocrinology,* 2014; 10(6): 338–351. https://doi.org/10.1038/nrendo.2014.51

[40] Bradley C, Harrison JE. (2007). Fall-related hospitalisations among older people: Sociocultural and regional aspects. *AIHW*. Available at: https://www.aihw.gov.au/getmedia/17a9a4b4-32a1-4481-8a42-b67ec21db92f/10447.pdf.aspx?inline=true, accessed on 28th March 2020

[41] Wolff J, McCrone P, Patel A, Kaier K, Normann C. Predictors of length of stay in psychiatry: Analyses of electronic medical records. *BMC Psychiatry*, 2015;*15*(1): 238. https://doi.org/10.1186/s12888-015-0623-6

[42] Stubbs B, Patchay B, Soundy A, Schofield P. The avoidance of activities due to fear of

For peer review only

For peer review only - http://bmjopen.bmj.com/site/about/guidelines.xhtml

falling contribute to sedentary behavior among community-dwelling older adults with chronic musculoskeletal pain: a multisite observational study. Pain Med, 2014; 15(11): 1861-1871. doi: 10.1111/pme.12570.

[43] Vestergaard P. Skeletal effects of central nervous system active drugs: anxiolytics, sedatives, antidepressants, lithum and neuroletpics. Curr Drug Saf, 2008; 3: 185-189. DOI : 10.2174/157488608785699432

[44] Zamani A, Omrani GR, Nasab MM. Lithium's effect on bone mineral density. Bone, 2009; 44: 331-334. doi: 10.1016/j.bone.2008.10.001.

[45] Klibanski A, Biller BMK, Rosenthal DI, Schoenfeld DA, Saxe V. Effects of prolactin and estrogen deficiency in amenorrheic bone loss. *Journal of Clinical Endocrinology and Metabolism*, 1988; *67*: 124–130. https://doi.org/10.1210/jcem-67-1-124

[46] Crews MPK, Howes OD. Is antipsychotic treatment linked to low bone mineral density and osteoporosis? A review of the evidence and the clinical implications. In *Human Psychopharmacology* 2012; 27(1): 15–23. https://doi.org/10.1002/hup.1265

[47] Coupland C, Dhiman P, Morriss R, Arthur A, Barton G, Hippisley-Cox J. Antidepressant use and risk of adverse outcomes in older people: Population based cohort study. *BMJ (Online)*, 2011; *343*: 4551. https://doi.org/10.1136/bmj.d4551

[48] Rauma PH, Honkanen RJ, Williams LJ, Tuppurainen MT, Kröger HP, Koivumaa-Honkanen H. Effects of antidepressants on postmenopausal bone loss - A 5-year longitudinal study from the OSTPRE cohort. *Bone*, 2016; *89*: 25–31. https://doi.org/10.1016/j.bone.2016.05.003

[49] Ruths S, Bakken MS, Ranhoff AH, Hunskaar S, Engesæter LB, Engeland A. Risk of hip fracture among older people using antihypertensive drugs: A nationwide cohort study.

For peer review only

BMC Geriatrics, 2015; *15*: 153. https://doi.org/10.1186/s12877-015-0154-5

[50] Rossini M, Viapiana O, Adami S, Idolazzi L, Buda S, Veronesi C, Degli Esposti L, Gatti D. Medication use before and after hip fracture: A population-based cohort and case-control study. *Drugs and Aging*, 2014; *31*: 547–553. https://doi.org/10.1007/s40266-014-0184-2

[51] Vestergaard P, Rejnmark L, Mosekilde L. Fracture risk associated with the use of morphine and opiates. *Journal of Internal Medicine*, 2006; *260*(1): 76–87. https://doi.org/10.1111/j.1365-2796.2006.01667.x

[52] Solomon DH, Mogun H, Garneau K, Fischer MA. Risk of fractures in older adults using antihypertensive medications. *Journal of Bone and Mineral Research*, 2011; *26*(7): 1561–1567. https://doi.org/10.1002/jbmr.356

[53] Pérez-Castrillón JL, Silva J, Justo I, Sanz A, Martín-Luquero M, Igea R, et al. Effect of quinapril, quinapril-hydrochlorothiazide, and enalapril on the bone mass of hypertensive subjects: Relationship with angiotensin converting enzyme polymorphisms. *American Journal of Hypertension*, 2003; *16*: 453–459. https://doi.org/10.1016/S0895-7061(03)00845-8

[54] Choi EJI, Kim SA, Kim NRI, Rhee JA, Yun YW, Shin MH. Risk factors for falls in older Korean adults: the 2011 Community Health Survey. *Journal of Korean Medical Science*, 2014; *29*(11): 1482–1487. https://doi.org/10.3346/jkms.2014.29.11.1482

[55] Menant JC, Wong AKW, Trollor JN, Close JCT, Lord SR. Depressive Symptoms and Orthostatic Hypotension Are Risk Factors for Unexplained Falls in Community-Living Older People. *Journal of the American Geriatrics Society*, 2016; *64*: 1073–1078. https://doi.org/10.1111/jgs.14104

For peer review only

[56] Soliman Y, Meyer R, Baum N. (2016). Falls in the Elderly Secondary to Urinary Symptoms. *Reviews in Urology*, 2016; *18*(1): 28–32.

[57] Wang J, You W, Jing Z, Wang R, Fu Z, Wang Y. Increased risk of vertebral fracture in patients with diabetes: a meta-analysis of cohort studies. In *International Orthopaedics* 2016; 40(6): 1299–1307. https://doi.org/10.1007/s00264-016-3146-y

[58] Ojike N, Sowers JR, Seixas A, Ravenell J, Rodriguez-Figueroa G, Awadallah M, et al. Psychological distress and hypertension: results from the national health interview survey for 2004-2013. *CardioRenal Medicine*, 2016; *6*(3): 198–208. https://doi.org/10.1159/000443933

[59] Friedman SM, Mendelson DA. Epidemiology of fragility fractures. *Clinics in Geriatric Medicine* 2014; 30(2): 175–181. https://doi.org/10.1016/j.cger.2014.01.001

[60] Carbone LD, Bková P, Fink HA, Lee JS, Chen Z, Ahmed A, Parashar S, Robbins JR. Hip fractures and heart failure: Findings from the Cardiovascular Health Study. *European Heart Journal*, 2010; *31*: 77–84. https://doi.org/10.1093/eurheartj/ehp483

[61] Moya A, Sutton R, Ammirati F, Blanc JJ, Brignole M, Dahm JB, et al. Guidelines for the diagnosis and management of syncope (version 2009). *European Heart Journal 2009;* 30(21): e2631–e2671. https://doi.org/10.1093/eurheartj/ehp298

[62] López-Ibarra PJ, Pastor MMC, Escobar-Jiménez F, Pardo MDS, GonzÍez AG, De Dios Luna J, et al. Bone mineral density at time of clinical diagnosis of adult-onset type 1 diabetes mellitus. *Endocrine Practice*, 2001; *7*: 346–351. https://doi.org/10.4158/EP.7.5.346

[63] Kim S-Y, Lee J-K, Sim S, Choi HG. Hearing impairement increases the risk of distal radius, hip and spine fractures: a longitudinal follow-up study using a national sample

For peer review only

cohort. PLoS One, 2018; 13(2): e0192820. doi: 10.1371/journal.pone.0192820

[64] Keller BK, Morton JL, Thomas VS, Potter JF. The effect of hearing and visual impairments on functional status. J Am Geriatr Soc, 1999; 47(11): 1319-1325. https://doi.org/10.1111/j.1532-5415.1999.tb07432.x

[65] American Geriatrics Society, British Geriatrics Society, and A. A. of, & Orthopaedic Surgeons. Guideline for the Prevention of Falls in Older Persons and American Academy of Orthn of opaedic Surgeons Panel on Falls Prevention. *American Geriatrics Society*, 2001; *49*(5): 664–672.

[66] Briggs R, Kennelly SP, Kenny RA. Does baseline depression increase the risk of unexplained and accidental falls in a cohort of community-dwelling older people? Data from The Irish Longitudinal Study on Ageing (TILDA). *International Journal of Geriatric Psychiatry*, 2018; *33*(2): e205–e211. https://doi.org/10.1002/gps.4770

[67] Van Den Berg M, Verdijk NA, Leusink GL, Wijnands-Van Gent CJM., Romeijnders AC, Pop VJM, et al. Depression after low-energy fracture in older women predicts future falls: A prospective observational study. *BMC Geriatrics*, 2011; *11*: 73. https://doi.org/10.1186/1471-2318-11-73

[68] Lee SC, Hu LY, Huang MW, Shen CC, Huang WL, Lu T, Hsu CL, Pan CC. Risk of vertebral fracture in patients diagnosed with a depressive disorder: A nationwide population-based cohort study. *Clinics*, 2017; *72*(1): 44–50. https://doi.org/10.6061/clinics/2017(01)08

[69] Gleason LJ, Menzies IB, Mendelson DA, Kates SL, Friedman SM. Diagnosis and Treatment of Osteoporosis in High-Risk Patients Prior to Hip Fracture. *Geriatric Orthopaedic Surgery & Rehabilitation*, 2012; *3*(2): 79–83. https://doi.org/10.1177/2151458512454878

For peer review only

For peer review only - http://bmjopen.bmj.com/site/about/guidelines.xhtml

[70] Appleby PN, Allen NE, Roddam AW, Key TJ. Physical activity and fracture risk: A prospective study of 1898 incident fractures among 34 696 British men and women. *Journal of Bone and Mineral Metabolism*, 2008; *26*: 191–198. https://doi.org/10.1007/s00774-007-0806-4

[71] Choi M, Hector M. Effectiveness of intervention programs in preventing falls: A systematic review of recent 10 years and meta-analysis. *Journal of the American Medical Directors Association*, 2012; *13*(2): e13–e21. https://doi.org/10.1016/j.jamda.2011.04.022

[72] Krogh J, Nordentoft M, Sterne JAC, Lawlor DA. The effect of exercise in clinically depressed adults: Systematic review and meta-analysis of randomized controlled trials. *Journal of Clinical Psychiatry*, 2011; *72*: 529–538. https://doi.org/10.4088/JCP.08r04913blu

[73] Zijlstra GAR, Van Haastregt JCM, Ambergen T, Van Rossum E, Van Eijk JTM, Tennstedt SL, Kempen GIJM. Effects of a multicomponent cognitive behavioral group intervention on fear of falling and activity avoidance in community-dwelling older adults: Results of a randomized controlled trial. *Journal of the American Geriatrics Society*, 2009; *57*(11): 2020–2028. https://doi.org/10.1111/j.1532-5415.2009.02489.x

For peer review only

For peer review only - http://bmjopen.bmj.com/site/about/guidelines.xhtml

For peer review only

For peer review only - http://bmjopen.bmj.com/site/about/guidelines.xhtml

Table 1: Characteristics of those Mood [affective] disorders patients who were admitted to hospital with a fall/fracture after a diagnosis of Mood [affective] disorders (F30* to F34*).

	Presence of falls		Presence of fractures	
**Characteristics of sample**	**No (n= 34,270)**	**Yes (n= 1,831)**	**No (n= 33,908)**	**Yes (n= 2,193)**
**Age at the time of Mood [affective] disorders diagnosis**				
18- 34	12685 (37.0)	205 (11.2)	12572 (37.1)	318 (14.5)
35- 49	11409 (33.3)	336 (18.4)	11346 (33.5)	399 (18.2)
50- 64	5829 (17.0)	362 (19.8)	5751 (17.0)	440 (20.1)
65- 79	2797 (8.2)	485 (26.5)	2728 (8.0)	554 (25.3)
80 & over	1550 (4.5)	443 (24.2)	1511 (4.5)	482 (22.0)
**Gender**				
Female	20627 (60.2)	1107 (60.5)	20348 (60.0)	1386 (63.2)
Male	13638 (39.8)	724 (39.5)	13555 (40.0)	807 (36.8)
**Ethnicity**				
White	20772 (60.6)	1519 (83.0)	20536 (60.6)	1755 (80.0)
Non-white	12450 (36.3)	288 (15.7)	12333 (36.4)	405 (18.5)
**Marital status**				
Cohabiting	7957 (23.2)	416 (22.7)	7867 (23.2)	506 (23.1)
Non-cohabiting	23028 (67.2)	1345 (73.5)	22776 (67.2)	1597 (72.8)
**Index of multiple deprivation: IMD 2015 (SD)**	27.97 (11.31)	27.90 (11.53)	27.97 (11.30)	27.97 (11.52)
Least deprived quintile	6657 (19.4)	388 (21.2)	6593 (19.4)	452 (20.6)
2nd most deprived quintile	6682 (19.5)	378 (20.6)	6643 (19.6)	417 (19.0)
3rd most deprived quintile	6706 (19.6)	354 (19.3)	6637 (19.6)	423 (19.3)
Most deprived quintile	6708 (19.6)	346 (18.9)	6612 (19.5)	442 (20.2)

For peer review only - http://bmjopen.bmj.com/site/about/guidelines.xhtml

**Medication prescription (within 6 months before or after Mood [affective] disorders diagnosis)**				
Anticholinergics	17714 (51.7)	1089 (59.5)	17489 (51.6)	1314 (59.9)
Antihypertensives	2561 (7.5)	386 (21.1)	2553 (7.5)	394 (18.0)
Antidepressants	19351 (56.5)	1168 (63.8)	19113 (56.4)	1406 (64.1)
Antipsychotics	8777 (25.6)	469 (25.6)	8694 (25.6)	552 (25.2)
Anxiolytics and Hypnotics	9443 (27.6)	558 (30.5)	9349 (27.6)	652 (29.7)
Analgesics	3083 (9.0)	405 (22.1)	3059 (9.0)	429 (19.6)
**Number of medications received (within 6 months before or after Mood [affective] disorders diagnosis)**				
0	10207 (29.8)	394 (21.5)	10126 (29.9)	475 (21.7)
1	5330 (15.6)	259 (14.1)	5275 (15.6)	314 (14.3)
2	7062 (20.6)	360 (19.7)	6961 (20.5)	461 (21.0)
3	6599 (19.3)	361 (19.7)	6503 (19.2)	457 (20.8)
4	3858 (11.3)	304 (16.6)	3839 (11.3)	323 (14.7)
5	1038 (3.0)	121 (6.6)	1029 (3.0)	130 (5.9)
6	176 (0.5)	32 (1.7)	175 (0.5)	33 (1.5)
**Other psychiatric conditions (within six months before or after Mood [affective] disorders diagnosis)**				
F00- F03 (Dementia)	754 (2.2)	207 (11.3)	749 (2.2)	212 (9.7)
F 20- F29 (Schizophrenia spectrum disorder)	2701 (7.9)	119 (6.5)	2676 (7.9)	144 (6.6)
F30- F31 (Bipolar affective disorder)	5489 (16.0)	222 (12.1)	5475 (16.1)	236 (10.8)
F50 (Eating Disorders)	447 (1.3)	13 (0.7)	441 (1.3)	19 (0.9)
F40- F48 (Neurotic, stress-related and somatoform disorders)	3652 (10.7)	147 (8.0)	3601 (10.6)	198 (9.0)
F 60 (Disorders of adult personality and behaviour)	1436 (4.2)	77 (4.2)	1428 (4.2)	85 (3.9)
**Problem HoNOS (score 2 or over) (within six months before or after Mood [affective] disorders diagnosis)**				
Agitated Behaviour	3571 (10.4)	234 (12.8)	3547 (10.5)	258 (11.8)

For peer review only - http://bmjopen.bmj.com/site/about/guidelines.xhtml

Self-Injury	3586 (10.5)	186 (10.2)	3545 (10.5)	227 (10.4)
Problem Drinking Drugs	2957 (8.6)	204 (11.1)	2932 (8.6)	229 (10.4)
Cognitive Problems	2821 (8.2)	349 (19.1)	2792 (8.2)	378 (17.2)
Physical Illness	6514 (19.0)	770 (42.1)	6355 (18.7)	929 (42.4)
Hallucinations	3198 (9.3)	175 (9.6)	3176 (9.4)	197 (9.0)
Depressed Mood	14582 (42.6)	766 (41.8)	14390 (42.4)	958 (43.7)
Relationship Problems	7644 (22.3)	342 (18.7)	7560 (22.3)	426 (19.4)
Daily Living Problems	5854 (17.1)	576 (31.5)	5723 (16.9)	707 (32.2)
Living Conditions Problems Score	3431 (10)	190 (10.4)	3404 (10.0)	217 (9.9)
Occupational Problems	5574 (16.3)	392 (21.4)	5513 (16.3)	453 (20.7)
Mean overall HoNoS score (SD)	10.67 (5.63)	11.64 (5.53)	10.64 (5.63)	11.91 (5.46)
Number with missing HoNoS	12545 (36.6)	523 (28.6)	12411 (36.6)	657 (30.0)
**Hospital admissions (within six months before or after Mood [affective] disorders diagnosis)**				
Ischaemia +CHD+ IHD	1128 (3.3)	210 (11.5)	1089 (3.2)	249 (11.4)
Arrhythmia + AF	1003 (2.9)	197 (10.8)	967 (2.9)	233 (10.6)
Heart failure	437 (1.3)	88 (4.8)	417 (1.2)	108 (4.9)
Diabetes	1516 (4.4)	264 (14.4)	1488 (4.4)	292 (13.3)
Hypotension	435 (1.3)	104 (5.7)	424 (1.3)	115 (5.2)
Hypercholesterolemia	1097 (3.2)	221 (12.1)	1090 (3.2)	228 (10.4)
Hypertension	2837 (8.3)	546 (29.8)	2752 (8.1)	631 (28.8)
Urinary tract infections (UTI)	1366 (4.0)	324 (17.7)	1297 (3.8)	393 (17.9)
Osteoporosis	409 (1.2)	121 (6.6)	183 (0.5)	347 (15.8)
Visual Disturbance and Blindness	241 (0.7)	53 (2.9)	246 (0.7)	48 (2.2)
Hearing Loss	189 (0.6)	41 (2.2)	174 (0.5)	56 (2.6)
Syncope or Collapse	1990 (5.8)	444 (24.2)	2022 (6.0)	412 (18.8)
Parkinson's Disease	116 (0.3)	45 (2.5)	128 (0.4)	33 (1.5)
Falls before diagnosis	750 (2.2)	267 (14.6)	749 (2.2)	268 (12.2)

For peer review only

For peer review only - http://bmjopen.bmj.com/site/about/guidelines.xhtml

Fractures before diagnosis	1075 (3.1)	231 (12.6)	860 (2.5)	446 (20.3)
mean number of attendances to A&E following Mood [affective] disorders diagnosis (SD)	3.94 (9.98)	16.64 (28.88)	4.00 (9.62)	13.67 (29.35)

For peer review only

Table 2: Univariate Cox proportional hazard model (95% CI) showing factors affecting time to first fall/fracture hospital admission since diagnosis of Mood [affective] disorders

	Outcome falls		Outcome fractures	
**Characteristics**	**HR (95% CI)**	**P value**	**HR (95% CI)**	**P value**
**Age at the time of Mood [affective] disorders diagnosis**				
18- 34	Ref.		Ref.	
35- 49	1.74 (1.46, 2.07)	**<0.001**	1.33 (1.15, 1.54)	**<0.001**
50- 64	3.88 (3.27, 4.61)	**<0.001**	3.03 (2.62, 3.50)	**<0.001**
65- 79	12.41 (10.54, 14.62)	**<0.001**	9.11 (7.94, 10.46)	**<0.001**
80 & over	26.94 (22.8, 31.83)	**<0.001**	18.46 (16.00, 21.29)	**<0.001**
Female gender	0.99 (0.90, 1.09)	0.87	1.12 (1.03, 1.22)	**0.01**
Non-European ethnicity	0.32 (0.28, 0.37)	**<0.001**	0.39 (0.35, 0.44)	**<0.001**
Non-cohabiting marital status	1.08 (0.97, 1.21)	0.17	1.05 (0.95, 1.16)	0.31
**Deprivation quintile (IMD 2015)**				
Least deprived quintile	Ref.		Ref.	
2nd least deprived quintile	0.83 (0.71, 0.96)	**0.01**	0.89 (0.78, 0.99)	**0.04**
3rd most deprived quintile	0.88 (0.76, 1.02)	**0.09**	0.90 (0.79, 1.03)	0.13
2nd most deprived quintile	0.94 (0.82, 1.09)	0.43	0.89 (0.78, 1.00)	**0.05**
Most deprived quintile	0.86 (0.74, 0.99)	**0.04**	0.94 (0.82, 1.07)	0.35
**Medication prescribed (within one year before or after Mood [affective] disorders diagnosis)**				
Anticholinergics received	1.42 (1.3, 1.56)	**<0.001**	1.45 (1.33, 1.57)	**<0.001**
Antihypertensive received	3.69 (3.3, 4.13)	**<0.001**	2.98 (2.67, 3.33)	**<0.001**
Antidepressants received	1.41 (1.29, 1.56)	**<0.001**	1.43 (1.31, 1.56)	**<0.001**

For peer review only - http://bmjopen.bmj.com/site/about/guidelines.xhtml

Antipsychotics received	0.95 (0.86, 1.06)	0.35	0.93 (0.84, 1.02)	0.12
Anxiolytics and Hypnotics received	1.11 (1.00, 1.22)	**0.05**	1.06 (0.97, 1.17)	0.18
Analgesics received	2.97 (2.66, 3.32)	**<0.001**	2.52 (2.27, 2.80)	**<0.001**
Increase in one type of polypharmacy	1.21 (1.18, 1.25)	**<0.001**	1.18 (1.15, 1.21)	**<0.001**
**Presence of other psychiatric conditions (within one year before or after Mood [affective] disorders diagnosis)**				
F00- F03 (Dementia)	6.90 (5.97, 7.98)	**<0.001**	5.65 (4.90, 6.51)	**<0.001**
F20- F29 (Schizophrenia spectrum disorder)	0.75 (0.62, 0.91)	**<0.001**	0.76 (0.64, 0.90)	**0.01**
F30- F31 (Bipolar affective disorder)	0.71 (0.62, 0.82)	**<0.001**	0.61 (0.54, 0.71)	**<0.001**
F40- F48 (Neurotic, stress-related and somatoform disorders)	0.76 (0.64, 0.90)	**<0.001**	0.86 (0.75, 0.99)	**0.05**
F50 (Eating Disorders)	0.53 (0.31, 0.91)	**0.02**	0.64 (0.41, 1.01)	0.06
F 60 (Disorders of adult personality and behaviour)	0.95 (0.76, 1.20)	0.68	0.87 (0.7, 1.09)	0.22
**Problem in HoNoS (scored 2 or more)**				
Agitated Behaviour	1.11 (0.97, 1.28)	0.14	1.03 (0.90, 1.17)	0.71
Self-Injury	0.84 (0.72, 0.98)	**0.02**	0.87 (0.76, 1.01)	0.06
Problem Drinking Drugs	1.11 (0.96, 1.29)	0.17	1.05 (0.91, 1.21)	0.48
Cognitive Problems	2.66 (2.35, 3.00)	**<0.001**	2.37 (2.11, 2.66)	**<0.001**
Physical Illness	3.88 (3.48, 4.34)	**<0.001**	4.17 (3.77, 4.63)	**<0.001**
Hallucinations	0.87 (0.74, 1.02)	0.08	0.82 (0.71, 0.96)	**0.01**
Depressed Mood	0.75 (0.67, 0.83)	**<0.001**	0.88 (0.80, 0.98)	**0.02**
Relationship Problems	0.65 (0.58, 0.74)	**<0.001**	0.71 (0.63, 0.79)	**<0.001**
Daily Living Problems	2.41 (2.16, 2.69)	**<0.001**	2.61 (2.36, 2.89)	**<0.001**
Living Conditions Problems	0.93 (0.80, 1.08)	0.34	0.89 (0.77, 1.02)	0.10
Occupational Problems	1.30 (1.15, 1.47)	**<0.001**	1.30 (1.16, 1.45)	**<0.001**
Overall increase in one unit of HoNoS	1.04 (1.03, 1.05)	**<0.001**	1.04 (1.04, 1.05)	**<0.001**
**Admitted to general hospital (within one year before or after Mood [affective] disorders diagnosis)**				

For peer review only - http://bmjopen.bmj.com/site/about/guidelines.xhtml

Ischaemia +CHD+ IHD	5.25 (4.54, 6.06)	**<0.001**	5.17 (4.53, 5.90)	**<0.001**
Arrhythmia + AF	6.30 (5.43, 7.31)	**<0.001**	6.10 (5.32, 6.99)	**<0.001**
Heart failure	7.81 (6.29, 9.68)	**<0.001**	7.89 (6.49, 9.59)	**<0.001**
Diabetes	4.66 (4.09, 5.31)	**<0.001**	4.18 (3.69, 4.73)	**<0.001**
Hypotension	7.29 (5.98, 8.90)	**<0.001**	6.51 (5.39, 7.86)	**<0.001**
Hypercholesterolemia	5.14 (4.47, 5.92)	**<0.001**	4.24 (3.70, 4.87)	**<0.001**
Hypertension	6.08 (5.50, 6.72)	**<0.001**	5.78 (5.26, 6.34)	**<0.001**
Urinary tract infections (UTI)	7.24 (6.42, 8.17)	**<0.001**	7.36 (6.59, 8.21)	**<0.001**
Osteoporosis	7.75 (6.44, 9.32)	**<0.001**	36.39 (32.35, 40.94)	**<0.001**
Visual Disturbance and Blindness	5.25 (3.99, 6.90)	**<0.001**	3.78 (2.84, 5.03)	**<0.001**
Hearing Loss	5.57 (4.09, 7.60)	**<0.001**	6.45 (4.95, 8.41)	**<0.001**
Syncope or Collapse	6.17 (5.54, 6.86)	**<0.001**	4.27 (3.84, 4.76)	**<0.001**
Parkinson's Disease	9.83 (7.31, 13.23)	**<0.001**	5.60 (3.97, 7.89)	**<0.001**
Falls before Mood [affective] disorders diagnosis	8.88 (7.8, 10.12)	**<0.001**	7.03 (6.18, 7.99)	**<0.001**
Fractures before Mood [affective] disorders diagnosis	5.31 (4.62, 6.10)	**<0.001**	10.67 (9.61, 11.85)	**<0.001**
Increase in one attendance to A&E following Mood [affective] disorders diagnosis	1.01 (1.01, 1.01)	**<0.001**	1.01 (1.01, 1.01)	**<0.001**

For peer review only

Table 3: Two models showing predictors of first fall hospital admission among Mood [affective] disorders. (used stepwise removal of factors that were not significant at 0.05 P value).

	Falls			
	Model 1 (n=20,938)		Model 2 (n=22,414)	
**Characteristics**	**HR (95% CI)**	**P value**	**HR (95% CI)**	**P value**
**Age at the time of Mood [affective] disorders diagnosis**				
18- 34	Ref.		Ref.	
35- 49	1.59 (1.23, 2.06)	**<0.001**	1.58 (1.24, 2.02)	**<0.001**
50- 64	3.11 (2.40, 4.01)	**<0.001**	2.98 (2.33, 3.81)	**<0.001**
65- 79	8.07 (6.31, 10.33)	**<0.001**	7.65 (6.07, 9.64)	**<0.001**
80 & over	12.40 (9.51, 16.17)	**<0.001**	11.80 (9.25, 15.05)	**<0.001**
Non-European ethnicity	0.43 (0.36, 0.51)	**<0.001**	0.45 (0.38, 0.52)	**<0.001**
Deprivation quintile (IMD 2015)				
Least deprived quintile	Ref.			
2nd least deprived quintile	0.99 (0.83, 1.19)	0.92		
3rd most deprived quintile	1.14 (0.95, 1.36)	0.17		
2nd most deprived quintile	1.15 (0.96, 1.38)	0.13		
Most deprived quintile	1.00 (0.84, 1.20)	0.85		
**Medication prescribed (within one year before or after diagnosis of Mood [affective] disorders)**				
Anticholinergics received	0.95 (0.74, 1.22)	0.68		
Antihypertensive received	0.98 (0.79, 1.21)	0.82		
Antidepressants received	0.91 (0.74, 1.11)	0.36		
Anxiolytics and Hypnotics received	1.00 (0.80, 1.24)	0.97		
Analgesics received	1.36 (1.10, 1.68)	**0.01**	1.39 (1.22, 1.58)	**<0.001**

For peer review only - http://bmjopen.bmj.com/site/about/guidelines.xhtml

Increase in one type of polypharmacy	1.02 (0.87, 1.19)	0.80		
**Presence of other psychiatric conditions (within one year before or after diagnosis of Mood [affective] disorders)**				
F00- F03 (Dementia)	1.05 (0.86, 1.27)	0.64		
F20- F29 (Schizophrenia spectrum disorder)	1.07 (0.85, 1.34)	0.57		
F30-F31 (Bipolar affective disorder)	0.87 (0.72, 1.06)	0.16		
F40- F48 (Neurotic, stress-related and somatoform disorders)	0.74 (0.60, 0.91)	**0.01**	0.75 (0.61, 0.91)	**0.01**
F50 (Eating Disorders)	0.86 (0.36, 2.09)	0.74		
**One unit increase in HoNoS**				
Self-Injury	1.10 (0.92, 1.32)	0.30		
Cognitive Problems	1.25 (1.06, 1.46)	**0.01**	1.28 (1.12, 1.46)	**<0.001**
Physical Illness	1.23 (1.07, 1.42)	**<0.001**	1.25 (1.10, 1.42)	**<0.001**
Depressed Mood	0.80 (0.70, 0.92)	**<0.001**	0.83 (0.74, 0.93)	**<0.001**
Relationship Problems	0.98 (0.84, 1.14)	0.77		
Daily Living Problems	1.01 (0.87, 1.17)	0.94		
Occupational Problems	0.90 (0.78, 1.03)	0.13		
**Admitted to general hospital (within one year before or after diagnosis of Mood [affective] disorders)**				
Ischaemia +CHD+ IHD	0.95 (0.78, 1.15)	0.59		
Arrhythmia + AF	0.88 (0.73, 1.07)	0.20		
Heart failure	1.37 (1.05, 1.78)	**0.02**	1.27 (1.00, 1.63)	**0.05**
Diabetes	1.37 (1.16, 1.63)	**<0.001**	1.36 (1.16, 1.59)	**<0.001**
Hypotension	1.38 (1.09, 1.75)	**0.01**	1.34 (1.07, 1.68)	**0.01**
Hypercholesterolemia	1.16 (0.96, 1.39)	0.13		
Hypertension	1.05 (0.88, 1.23)	0.63		
Urinary tract infections (UTI)	1.33 (1.13, 1.56)	**<0.001**	1.33 (1.14, 1.54)	**<0.001**

For peer review only - http://bmjopen.bmj.com/site/about/guidelines.xhtml

Osteoporosis	1.41 (1.08, 1.83)	**0.01**	1.37 (1.11, 1.70)	**<0.001**
Visual Disturbance and Blindness	1.22 (0.86, 1.71)	0.26		
Hearing Loss	0.91 (0.64, 1.30)	0.61		
Syncope or Collapse	1.94 (1.71, 2.26)	**<0.001**	2.05 (1.79, 2.36)	**<0.001**
Parkinson's Disease	1.25 (0.89, 1.77)	0.20		
Falls before Mood [affective] disorders diagnosis	1.81 (1.51, 2.18)	**<0.001**	1.81 (1.54, 2.13)	**<0.001**
Fractures before Mood [affective] disorders diagnosis	0.98 (0.78, 1.22)	0.86		
Increase in one attendance to A&E following Mood [affective] disorders * diagnosis	1.01 (1.01, 1.01)	**<0.001**	1.01 (1.01, 1.01)	**<0.001**

For peer review only - http://bmjopen.bmj.com/site/about/guidelines.xhtml

Table 4: Two models showing predictors of first fracture hospital admission among Mood [affective] disorders patients.

	Model 1 (n=20,936)		Model 2 (n=22,322)	
**Characteristics**	**HR (95% CI)**	**P value**	**HR (95% CI)**	**P value**
**Age at the time of Mood [affective] disorders diagnosis**				
18- 34	Ref.		Ref.	
35- 49	1.27 (1.02, 1.57)	**0.03**	1.27 (1.03, 1.55)	**0.02**
50- 64	2.31 (1.85, 2.88)	**<0.001**	2.28 (1.85, 2.81)	**<0.001**
65- 79	5.75 (4.65, 7.11)	**<0.001**	5.55 (4.54, 6.77)	**<0.001**
80 & over	7.46 (5.90, 9.45)	**<0.001**	7.37 (5.93, 9.16)	**<0.001**
Female gender	1.21 (1.08, 1.36)	**<0.001**	1.15 (1.03, 1.29)	**0.01**
Non-European ethnicity	0.59 (0.51, 0.68)	**<0.001**	0.59 (0.51, 0.67)	**<0.001**
Deprivation quintile (IMD 2015)				
Least deprived quintile	Ref.			
2nd least deprived quintile	1.07 (0.91, 1.28)	0.41		
3rd most deprived quintile	1.11 (0.94, 1.32)	0.23		
2nd most deprived quintile	1.17 (0.99, 1.39)	0.07		
Most deprived quintile	1.00 (0.85, 1.19)	0.96		
**Medication prescribed (within one year before or after diagnosis of Mood [affective] disorders)**				
Anticholinergics received	1.08 (0.85, 1.26)	0.76		
Antihypertensive received	0.76 (0.65, 0.91)	**<0.001**	0.80 (0.70, 0.92)	**<0.001**
Antidepressants received	1.08 (0.91, 1.28)	0.37		
Analgesics received	1.28 (1.08, 1.52)	**<0.001**	1.33 (1.17, 1.51)	**<0.001**

For peer review only - http://bmjopen.bmj.com/site/about/guidelines.xhtml

Increase in one type of polypharmacy	1.01 (0.93, 1.11)	0.75		
**Presence of other psychiatric conditions (within one year before or after diagnosis of Mood [affective] disorders)**				
F00- F03 (Dementia)	1.14 (0.96, 1.41)	0.12		
F20- F29 (Schizophrenia spectrum disorder)	1.11 (0.91, 1.38)	0.30		
F30- F31 (Bipolar affective disorder)	0.81 (0.67, 0.98)	**0.03**	0.80 (0.68, 0.95)	**0.01**
F40- F48 (Neurotic, stress-related and somatoform disorders)	0.82 (0.68, 0.99)	**0.04**	0.83 (0.70, 0.99)	**0.04**
**One unit increase in HoNoS**				
Cognitive Problems	1.02 (0.87, 1.19)	0.83		
Physical Illness	1.36 (1.19, 1.56)	**<0.001**	1.48 (1.31, 1.67)	**<0.001**
Hallucinations	0.91 (0.76, 1.10)	0.34		
Depressed Mood	0.83 (0.72, 0.94)	**0.01**	0.91 (0.82, 1.00)	**0.05**
Relationship Problems	0.89 (0.77, 1.02)	0.10		
Daily Living Problems	1.12 (0.98, 1.29)	0.11		
Occupational Problems	0.91 (0.79, 1.04)	0.15		
**Admitted to general hospital (within one year before or after diagnosis of Mood [affective] disorders)**				
Ischaemia +CHD+ IHD	1.01 (0.85, 1.22)	0.88		
Arrhythmia + AF	1.03 (0.86, 1.23)	0.78		
Heart failure	1.44 (1.12, 1.86)	**0.01**	1.43 (1.14, 1.80)	**<0.001**
Diabetes	1.22 (1.04, 1.44)	**0.02**	1.23 (1.06, 1.44)	**<0.001**
Hypotension	1.18 (0.93, 1.48)	0.17		
Hypercholesterolemia	0.85 (0.71, 1.03)	0.10		
Hypertension	1.05 (0.90, 1.23)	0.53		
Urinary tract infections (UTI)	1.49 (1.28, 1.74)	**<0.001**	1.52 (1.32, 1.75)	**<0.001**
Osteoporosis	7.31 (5.82, 9.18)	**<0.001**	7.00 (5.65, 8.70)	**<0.001**
Visual Disturbance and Blindness	1.06 (0.74, 1.53)	0.74		

For peer review only

Hearing Loss	1.40 (1.03, 1.92)	**0.03**	1.46 (1.09, 1.96)	**0.01**
Syncope or Collapse	1.18 (1.02, 1.36)	**0.03**		
Parkinson's Disease	0.85 (0.58, 1.25)	0.41		
Falls before Mood [affective] disorders diagnosis	1.02 (0.85, 1.22)	0.83		
Fractures before Mood [affective] disorders diagnosis	1.26 (1.01, 1.57)	**0.04**	1.33 (1.10, 1.63)	**<0.001**
Increase in one attendances to A&E following Mood [affective] disorders diagnosis	1.01 (1.01, 1.01)	**<0.001**	1.01 (1.01, 1.01)	**<0.001**

For peer review only

For peer review only

For peer review only - http://bmjopen.bmj.com/site/about/guidelines.xhtml

The RECORD statement – checklist of items, extended from the STROBE statement, that should be reported in observational studies using routinely collected health data.

	Item No.	STROBE items	Location in manuscript where items are reported	RECORD items	Location in manuscript where items are reported
**Title and abstract**					
	1	(a) Indicate the study's design with a commonly used term in the title or the abstract (b) Provide in the abstract an informative and balanced summary of what was done and what was found	Page 1 and 2	RECORD 1.1: The type of data used should be specified in the title or abstract. When possible, the name of the databases used should be included.<br><br>RECORD 1.2: If applicable, the geographic region and timeframe within which the study took place should be reported in the title or abstract.<br><br>RECORD 1.3: If linkage between databases was conducted for the study, this should be clearly stated in the title or abstract.	Page 2
**Introduction**					
Background rationale	2	Explain the scientific background and rationale for the investigation being reported	Page 4		
Objectives	3	State specific objectives, including any prespecified hypotheses	Page 4		
**Methods**					
Study Design	4	Present key elements of study design early in the paper	Page 5		
Setting	5	Describe the setting, locations, and relevant dates, including periods of recruitment, exposure, follow-up, and data collection	Page 5		
Participants	6	*(a) Cohort study* - Give the	Page 5	RECORD 6.1: The methods of study	Page 5

For peer review only - http://bmjopen.bmj.com/site/about/guidelines.xhtml

		eligibility criteria, and the sources and methods of selection [illegible]		population selection (such as codes or algorithms used to identify subjects) [illegible]	

Variables	7	Clearly define all outcomes, exposures, predictors, potential confounders, and effect modifiers. Give diagnostic criteria, if applicable.	Page 6-8	RECORD 7.1: A complete list of codes and algorithms used to classify exposures, outcomes, confounders, and effect modifiers should be provided. If these cannot be reported, an explanation should be provided.	Page 6-8
Data sources/ measurement	8	For each variable of interest, give sources of data and details of methods of assessment (measurement). Describe comparability of assessment methods if there is more than one group	Page 6-8		
Bias	9	Describe any efforts to address potential sources of bias	Page 9		
Study size	10	Explain how the study size was arrived at	Page 5		
Quantitative variables	11	Explain how quantitative variables were handled in the analyses. If applicable, describe which groupings were chosen, and why	Page 6-8		

For peer review only

Statistical methods	12	(a) Describe all statistical methods, including those used to control for confounding<br>(b) Describe any methods used to examine subgroups and interactions<br>(c) Explain how missing data were addressed<br>(d) *Cohort study* - If applicable, explain how loss to follow-up was addressed<br>*Case-control study* - If applicable, explain how matching of cases and controls was addressed<br>*Cross-sectional study* - If applicable, describe analytical methods taking account of sampling strategy<br>(e) Describe any sensitivity analyses	Page 9		
Data access and cleaning methods		..		RECORD 12.1: Authors should describe the extent to which the investigators had access to the database population used to create the study population.<br><br>RECORD 12.2: Authors should provide information on the data cleaning methods used in the study.	Page 5
Linkage		..		RECORD 12.3: State whether the	Page 6
				study included person-level, institutional-level, or other data linkage across two or more databases. The methods of linkage and methods of linkage quality evaluation should be provided.	
**Results**					

For peer review only

Participants	13	(a) Report the numbers of individuals at each stage of the study (*e.g.*, numbers potentially eligible, examined for eligibility, confirmed eligible, included in the study, completing follow-up, and analysed)<br>(b) Give reasons for non-participation at each stage.<br>(c) Consider use of a flow diagram	Page 9	RECORD 13.1: Describe in detail the selection of the persons included in the study (*i.e.,* study population selection) including filtering based on data quality, data availability and linkage. The selection of included persons can be described in the text and/or by means of the study flow diagram.	Page 9
Descriptive data	14	(a) Give characteristics of study participants (*e.g.*, demographic, clinical, social) and information on exposures and potential confounders<br>(b) Indicate the number of participants with missing data for each variable of interest<br>(c) *Cohort study* - summarise follow-up time (*e.g.*, average and total amount)	Table 1 and page 9		
Outcome data	15	*Cohort study* - Report numbers of outcome events or summary measures over time<br>*Case-control study* - Report numbers in each exposure category, or summary measures of exposure<br>*Cross-sectional study* - Report numbers of outcome events or	Page 10		
		summary measures			

For peer review only

Main results	16	(a) Give unadjusted estimates and, if applicable, confounder-adjusted estimates and their precision (e.g., 95% confidence interval). Make clear which confounders were adjusted for and why they were included<br>(b) Report category boundaries when continuous variables were categorized<br>(c) If relevant, consider translating estimates of relative risk into absolute risk for a meaningful time period	Page 10-11		
Other analyses	17	Report other analyses done—e.g., analyses of subgroups and interactions, and sensitivity analyses	Not applicable		
**Discussion**					
Key results	18	Summarise key results with reference to study objectives	Page 13-16		
Limitations	19	Discuss limitations of the study, taking into account sources of potential bias or imprecision. Discuss both direction and magnitude of any potential bias	Page 16-17	RECORD 19.1: Discuss the implications of using data that were not created or collected to answer the specific research question(s). Include discussion of misclassification bias, unmeasured confounding, missing data, and changing eligibility over time, as they pertain to the study being reported.	Page 16-17
Interpretation	20	Give a cautious overall interpretation of results considering objectives, limitations, multiplicity of analyses, results from similar studies, and other relevant evidence	Page 17-18		
Generalisability	21	Discuss the generalisability (external validity) of the study results	Page 17		

For peer review only

Other Information					
Funding	22	Give the source of funding and the role of the funders for the present study and, if applicable, for the original study on which the present article is based	Page 18		
Accessibility of protocol, raw data, and programming code		..		RECORD 22.1: Authors should provide information on how to access any supplemental information such as the study protocol, raw data, or programming code.	Page 19

*Reference: Benchimol EI, Smeeth L, Guttmann A, Harron K, Moher D, Petersen I, Sørensen HT, von Elm E, Langan SM, the RECORD Working Committee. The REporting of studies Conducted using Observational Routinely-collected health Data (RECORD) Statement. *PLoS Medicine* 2015; in press.

*Checklist is protected under Creative Commons Attribution (CC BY) license.

For peer review only

BMJ Open

Predictors of falls and fractures leading to hospitalisation in 36,101 people with affective disorders: A large representative cohort study

Journal:	*BMJ Open*
Manuscript ID	bmjopen-2021-055070.R1
Article Type:	Original research
Date Submitted by the Author:	02-Feb-2022
Complete List of Authors:	Ma, Ruimin; King's College London, Psychological Medicine<br>Perera, Gayan; King's College London, Psychological Medicine<br>Romano, Eugenia; King's College London, Psychological Medicine<br>Vancampfort, Davy; KU Leuven<br>Koyanagi, Ai; Parc Sanitari Sant Joan de Deu, Research and Development Unit<br>Stewart, Robert; King's College London, Institute of Psychiatry<br>Mueller, Christoph; King's College London, Department of Old Age Psychiatry<br>Stubbs, B; King's College London, Psychological Medicine
**Primary Subject Heading**:	Mental health
Secondary Subject Heading:	Epidemiology
Keywords:	MENTAL HEALTH, Anxiety disorders < PSYCHIATRY, Adult psychiatry < PSYCHIATRY, Depression & mood disorders < PSYCHIATRY

SCHOLARONE™
Manuscripts

I, the Submitting Author has the right to grant and does grant on behalf of all authors of the Work (as defined in the below author licence), an exclusive licence and/or a non-exclusive licence for contributions from authors who are: i) UK Crown employees; ii) where BMJ has agreed a CC-BY licence shall apply, and/or iii) in accordance with the terms applicable for US Federal Government officers or employees acting as part of their official duties; on a worldwide, perpetual, irrevocable, royalty-free basis to BMJ Publishing Group Ltd ("BMJ") its licensees and where the relevant Journal is co-owned by BMJ to the co-owners of the Journal, to publish the Work in this journal and any other BMJ products and to exploit all rights, as set out in our licence.

The Submitting Author accepts and understands that any supply made under these terms is made by BMJ to the Submitting Author unless you are acting as an employee on behalf of your employer or a postgraduate student of an affiliated institution which is paying any applicable article publishing charge ("APC") for Open Access articles. Where the Submitting Author wishes to make the Work available on an Open Access basis (and intends to pay the relevant APC), the terms of reuse of such Open Access shall be governed by a Creative Commons licence – details of these licences and which Creative Commons licence will apply to this Work are set out in our licence referred to above.

Other than as permitted in any relevant BMJ Author's Self Archiving Policies, I confirm this Work has not been accepted for publication elsewhere, is not being considered for publication elsewhere and does not duplicate material already published. I confirm all authors consent to publication of this Work and authorise the granting of this licence.

For peer review only

Predictors of falls and fractures leading to hospitalisation in 36,101 people with affective disorders: A large representative cohort study

Running title: Predictors of falls and fractures in people with affective disorders

Ruimin Ma[a^*], Gayan Perera^[a], Eugenia Romano[a], Davy Vancampfort[c, d], Ai Koyanagi[e, f], Robert Stewart[a, b], Christoph Mueller^^[a, b] Brendon Stubbs^^[a, b],

[a] Institute of Psychiatry, Psychology and Neuroscience (IoPPN), King's College London, London, United Kingdom

[b] South London and Maudsley NHS Foundation Trust, London, United Kingdom

[c] KU Leuven – University of Leuven, Department of Rehabilitation Sciences, Leuven, Belgium

[d] KU Leuven – University of Leuven, University Psychiatric Centre KU Leuven, Leuven, Kortenberg, Belgium

[e] Research and Development Unit, Parc Sanitari Sant Joan de Déu, Universitat de Barcelona, Fundació Sant Joan de Déu, Dr. Antoni Pujadas, 42 ,Sant Boi de Llobregat, Barcelona 08830, Spain

[f] ICREA, Pg. Lluis Companys 23, Barcelona, Spain

^ Joint first author

^^ Joint last author

- Corresponding author at: Psychological Medicine, Institute of Psychiatry, Psychology and Neuroscience (IoPPN), King's College London, De Crespigny Park, London, BOX SE5 8AF, United Kingdom.

  Email address: ruimin.1.ma@kcl.ac.uk

For peer review only

For peer review only - http://bmjopen.bmj.com/site/about/guidelines.xhtml

Abstract (257/300)

Objectives: To investigate predictors of falls and fractures leading to hospitalisation in people with affective disorders.

Design: Cohort study

Setting: the South London and Maudsley NHS Foundation Trust (SLaM) Biomedical Research Centre (BRC) Case Register

Participants: A large cohort of people with affective disorders (ICD10 codes F30-F34) diagnosed between January 2008 and March 2016 was assembled using data from the SLaM BRC Case Register

Primary and secondary outcome measures: Falls and fractures leading to hospitalisation were ascertained from linked national hospitalisation data. Multivariable Cox proportional hazards analyses were administrated to identify predictors of first falls and fractures.

Results: Of 36,101 people with affective disorders (mean age 44.4 years, 60.2% female), 816 (incidence rate 9.91 per 1000 person years) and 1,117 (incidence rate 11.92 per 1000 person years) experienced either a fall or fracture respectively. In multivariable analyses, older age, analgesics use, increased physical illness burden, previous hospital admission due to certain co-morbid physical illnesses and increase in attendances to accident and emergency services following diagnosis were significant risk factors for both falls and fractures. Having a history of falls was a strong risk factor for recurrent falls, and a previous fracture was also associated with future fractures.

Conclusions: Over a mean 5 years' follow-up, approximately 8% of people with affective disorders were hospitalised with a fall or fracture. Several similar factors were found to predict risk of falls and fracture, for example, older age, comorbid physical disorders and analgesic use. Routine screening for bone mineral density and falls prevention programmes should be considered for this clinical group.

For peer review only - http://bmjopen.bmj.com/site/about/guidelines.xhtml

Key words: osteoporosis, affective disorders, falls, fractures, hospital admission

Strength and limitation of the study:

- Predictors of falls and fractures leading to hospitalisation in people with affective disorders were investigated with a large representative cohort
- Data of the study were derived directly from the electronic health record
- Falls and fractures leading to hospitalisation were ascertained from linked national hospitalisation data
- We did not stratify according to different affective disorder subtypes and psychotropic medication categories
- We had no information on lifestyle factors and type of fractures recorded at admission

For peer review only

Word count: 3636/4000

Introduction

Falls and fractures are strongly related with increased morbidity, mortality, disability, and healthcare expenditure in the general population [1], and they are a frequent issue in older adults [2]. They often overlap, with more than 90% of hip fractures caused by falls [3] and share similar and multifactorial causes, such as muscle weakness [4] and use of antiepileptic drugs [5]. Several government strategies have been implemented to prevent and assess falls in the general population [6]. Fractures, for example, hip and spine fractures increase the risk of future falls and fractures [7]. Over 3 million people in the UK are at a high risk for fractures [8], and prevention has been acknowledged as a worldwide priority [9]. However, management is difficult, as causes for falls and fractures can be both intrinsic and extrinsic, and prevention requires both pharmacologic and nonpharmacologic interventions [3].

Despite some progress in the general population, there is poor evidence on risk factors for falls and fractures for patients with affective disorders. People with mental health problems like depression and schizophrenia report higher risks of falls and fractures compared to the general population [10, 11], and despite poor evidence in people with bipolar disorders, a few studies suggest this population may also be at increased risk [12, 13].

Several studies have investigated the association between bone loss and depression, but there is a lack of large-scale cohort studies focussing on falls and fractures and related predictors. Another major limitation is that the majority of studies have relied on self-reported affective symptoms rather than clinical diagnosis [14, 15].

Given the increased concerns about the multifactorial causes of falls and fractures [3, 4, 5], and as an answer to the lack of large-scale studies on the matter, this study examines a large representative cohort study to investigate predictors of falls and fractures leading to hospitalisation among people with clinically diagnosed affective disorders. In this way, we aim

For peer review only

to provide results based on clinical diagnosis rather than self-reported data [14, 15], presenting a solid overview on potential risk factors for falls and fractures in the high-risk population of people with affective disorders.

Methods

A retrospective observational study was carried out using data from the South London and Maudsley NHS Foundation Trust (SLaM) Biomedical Research Centre (BRC) Case Register. SLaM is one of the largest mental health and dementia care providers in Europe, serving a geographic catchment of four South London Boroughs (including Lambeth, Lewisham, Southwark, and Croydon) with a population in excess of 1.3 million. Data for this study were retrieved using the Clinical Record Interactive Search (CRIS) platform, which enables a de-identified version of SLaM's electronic health record to be accessible for research projects within a robust and patient-led government framework [16]. The SLaM BRC Case Register has been described in detail elsewhere [17] and has supported a wide range of studies [18, 19], including longitudinal cohort studies investigating falls and fractures in other populations [20, 21]. Data are currently archived in CRIS on more than 450,000 cases with a wide variety of mental disorders. CRIS has full approval for secondary analysis (Oxford Research Ethnic Committee C, reference 18/SC/0372). Data from source structured fields have been extensively supplemented through natural language process (NLP) applications using Generalised Architecture for Text Engineering software, applying information extraction techniques to derive structured information from the extensive text fields held in the mental health record [17].

Participants, study period and additional data sources

All SLaM patients with mood [affective] disorders (ICD10 codes F30-F34), diagnosed between 1st of January 2008 and 31st March 2016, were included. Those patients with an age

For peer review only - http://bmjopen.bmj.com/site/about/guidelines.xhtml

under 18 at the time of mood disorders were excluded. SLaM patient records have been linked with national Hospital Episode Statistics (HES) which are compiled from all NHS Trusts in England (both acute and mental health services), including statistical abstracts of records of all inpatient episodes, as well as outpatient and emergency care [17]. In addition, CRIS data have been linked to the Office for National Statistics (ONS) mortality records over the same period.

Patient and public involvement (PPI)

The entire research program was developed after extensive patient, public and carer input, who helped develop the study, and contributed to the funding applicable. They co-developed the research questions, outcomes and contributed to factors included in the models. Moreover, they reinforced this research as a neglected topic of utmost importance to them. The PPI group designed the study with the senior researcher of the paper (Dr BS), they were involved in the conception of the idea, the planning of the study during bimonthly meetings to discuss study design, hypotheses, outcomes and what matters to patients of interest. They were also involved in helping interpret the data outputs and publication. Results will be summarised and made available to the PPI group through local newsletters and inform clinical services and local co-developed Recovery College Courses.

Co-primary outcome: falls and any fractures

The co-primary outcomes were hospital admissions relating to a fall or any fracture extracted from linked HES data and all discharge diagnoses (primary or any secondary diagnosis codes) recorded between January 2008 to March 2016, and based on the following ICD 10 codes: i) falls (W00-W19); ii) fractures (M80-M84, M907, S02, S12, S32, S42, S52, S62, S72, S82, S92, S22, T02, T08, T10, T12X, T902, T911, T912, T921). In addition, linked mortality records from the ONS linkage were examined for any instance of fall or fracture ICD codes in

For peer review only - http://bmjopen.bmj.com/site/about/guidelines.xhtml

any cause of death field on the death certificate to identify the date of death attributed to a fall or fracture.

Measurements

Several additional measurements were obtained from CRIS. All independent variables (covariates) were defined according to the value closest to the date of the first recorded mood [affective] disorder diagnosis. Demographic covariates comprised: age at diagnosis, gender, and ethnicity [multiple codes categorised into 1) White, consists of White British, White Irish and other White; and 2) Non-White]. Index of Multiple Deprivation (IMD 2015) for the neighbourhood of residence (i.e. Lower Super Output Area, which consists of approximately 650 households) at the time of diagnosis was divided into quintiles according to deprivation scores with equal size allocated to each group. The IMD has previously been used in CRIS [22] and combines Census-derived data at area level across several domains including income, employment, health, education, barriers to housing and services, living environment, and crime [23]. Information on cohabiting status (Cohabiting: married/civil partner, married, cohabiting; Non-cohabiting: single, divorced, civil partnership dissolved, widowed, separated) was also ascertained at the index diagnosis.

Illness burden

The Health of the Nation Outcome Scales (HoNOS) [24] are routinely administered measures of illness burden in UK mental health services and are recorded in structured fields on the electronic health record. Individual HoNOS item scores (agitated behaviour, self-injury, problem drinking & drugs, cognitive problems, physical illness, hallucinations, depressed mood, relationship problems, daily living problems, living conditions problems, occupational problems) and dates were obtained within 6 months before or after the date of the index

For peer review only

diagnosis, and the closest scores in time to this date were included in analyses. Each HoNOS item is rated on a Likert Scale, ranging from 0 (i.e. no problem) to 4 (i.e. severe or very severe problem). A detailed glossary for HoNOS is reported elsewhere [25]. Scores 2 or over in the individual HoNOS scales were classified as having a problem on each item, generating binary covariates.

Mental disorder comorbidity

Diagnoses of F00-F03 (dementia); F20-F29 (schizophrenia spectrum disorder); F40-F48 (neurotic, stress-related and somatoform disorders); F50 (eating disorders); F60-F69 (disorders of adult personality and behaviour) were ascertained within one year before or after the index diagnosis of mood [affective] disorder.

Medication

Medications received were extracted from structured medication fields in the record, supplemented by an NLP application applied to text fields ascertaining mentions of current medication [17]. Presence or not of the following medication groups was ascertained on the basis of information within six months before or after the index diagnosis: anticholinergics, antihypertensives, antidepressants, antipsychotics, anxiolytics and hypnotics, and analgesics. The total number of medications prescribed for any condition was calculated for each participant and used as a continuous variable.

Physical comorbidity

Information on physical comorbidities was ascertained utilising the data linkage between CRIS and national HES records. Information on all ICD-10 diagnoses at discharge (primary or any secondary diagnosis codes) was ascertained from any hospitalisations within 6 months before or after the index diagnosis date and the following binary variables generated: i) Ischaemic

For peer review only

heart disease (IHD; I20, I21, I22, including coronary heart disease (CHD; I25); ii) Arrhythmia (I44-I49, including atrial fibrillation (AF; I48); iii) Heart failure (I50); iv) Diabetes (E08, E09, E10, E11, E12, E13; v) Hypotension (I95-99); vi) Hypercholesterolemia (E78); vii) Hypertension (I10-15); viii) Urinary tract infections (UTI) (N39); ix) Osteoporosis (M80-85); x) Visual disturbance and blindness (H53-54); xi) Hearing loss (H90-95); xii) Syncope or collapse (R50-R69); xiii) Parkinson's disease (G20). Furthermore, occurrence of falls and/or fractures before the index diagnosis was also collected. Finally, the number of attendances to accident and emergency services (A&E) following the index diagnosis was also recorded.

Statistical analysis

The study sample was described initially in terms of demographic and clinical variables, followed by unadjusted Cox proportional hazard models to predict the first fall and first fracture separately after the index diagnosis of mood [affective] disorder. The predictor variables at baseline used in the first univariate models included sociodemographic information (year of index diagnosis, mean age, gender, ethnicity, marital status), medications (antipsychotics, anxiolytics & hypnotics, antidepressants, analgesics, anticholinergics, antihypertensives), comorbid psychiatric diagnosis (F20-29 schizophrenia spectrum disorder, F40-48 neurotic/stress disorders, F00-F03 dementia, F50 eating disorders, F60 disorders of adult personality and behaviour), HONOS scores (mean total and each individual item) and physical health comorbidities (as indicated above). Factors that yielded a p-value < 0.10 in the univariate model for fall or fracture outcome were subsequently entered into the multivariable model. A final multivariable Cox proportional hazards model, using stepwise backward elimination technique where those variables not significant (p-value > 0.05) were eliminated, with hazard ratios and 95% confidence intervals (CI) displayed. Having checked a correlation matrix of coefficients in the Cox model, total number of medications received was substantially collinear

For peer review only

with individual types of medication received; therefore, this total number was removed as a covariate. All analyses were conducted utilising STATA, version 13.

Results

The sample comprised 36,101 people with a diagnosis of mood [affective] disorder (F30* - F34*) (mean age at first diagnosis 44.4, SD: 17.8, 60.2% female). Over a mean 5 years' follow-up, 2,948 patients had a fall and/or a fracture recorded: 816 only a fall, 1,117 only a fracture, and 1,015 both; 1,831 with any fall and 2,193 with any fracture (mean age at first fall 64.2, SD: 24.8; mean age at first fracture 62.5, SD: 22.8 (Supplementary material Table 1). The incidence rate of falls was 9.91 per 1,000 person years and that for fractures 11.92 per 1,000 person years. Table 1 summarises characteristics of those who had a recorded fall or fracture compared to those who did not.

[Table 1]

Length of hospital stay

The mean length of hospitalisation following a fall (n = 1,831) was 7.9 days (range 0-374), for a total of 20,767 full days in hospital. The mean length of stay in hospital following a fracture (n=2,193) was 13.2 days (range 0-374), for a total of 40,548 days. This equates to 18.51 years of inpatient hospital stay for 1,000-person years of follow-up due to a fall and 36.15 years of inpatient hospital stay for 1,000-person years of follow-up due to a fracture. For the 1,831 patients reporting a fall, the mean hospital admissions due to a fall was 1.5 (range 1- 15); for the 2,193 patients reporting a fracture, the mean number of hospital admissions due to a fracture was 2.3 (range 1-42).

For peer review only - http://bmjopen.bmj.com/site/about/guidelines.xhtml

Factors associated with falls and fractures

Cox proportional hazard models analysing unadjusted predictors of falls and fractures (95% CI) are reported in Table 2.

[Table 2]

Multivariable predictors of falls

Multivariable models of factors associated with first fall hospital admission are presented in Table 3. In Model 2, older age was strongly associated with higher risk, and Non-European ethnicity with lower risk, but neighbourhood deprivation reported no association. Analgesics had a significant association (with increased risk), and of co-morbid psychiatric conditions, only the ICD-10 F40-F48 group had a significant association (with reduced risk). Of the HoNOS items, cognitive problems and physical illness were associated with higher risk, and depressed mood problems with lower risk. Higher risk of falls was associated with several hospitalisation discharge diagnoses, namely heart failure, diabetes, hypotension, UTI, osteoporosis, and syncope or collapse. Hospitalised fall was associated with having a previous history of falls reported before the index diagnosis and with increased A&E attendance after the index diagnosis.

[Table 3]

Multivariable predictors of fractures

Table 4 presents multivariable models of factors associated with first fracture hospitalisation. Fracture risk was increased in older patients and women and was decreased in those of Non-European. Fracture risk was lower in patients receiving antihypertensives and higher in those receiving analgesics. It was also lower in those with neurotic, stress-related and somatoform

For peer review only - http://bmjopen.bmj.com/site/about/guidelines.xhtml

disorders (i.e. F40-F48), as well as bipolar affective disorder and manic episodes (i.e. F30-F31). An elevated fracture risk was associated with physical illness on the HoNOS. Fracture risk was independently predicted by previous hospitalisations for heart failure, diabetes, UTI, osteoporosis, hearing loss and preceding fracture-related (but not fall-related) hospitalisations. Fracture risk was also associated with higher levels of post-diagnostic A&E attendance.

[Table 4]

For peer review only

Discussion

To our knowledge, the current study is the first using representative data to investigate the predictors of hospitalised falls and fractures in people with clinically diagnosed affective disorders. Our data suggest that out of 36,101 people with affective disorders, 816 (i.e. 2.26%) and 1,117 (i.e. 3.09%) of patients experienced a fall and fracture, respectively. Length of hospital stay was considerable, equating to 18.51 and 36.15 years of inpatient hospital stay for 1,000-person years of follow-up due to a fall and a fracture, respectively. The key factors increasing the risk of a hospitalised fall were older age, analgesic use, increased illness burden due to cognitive problems and physical illness, a history of general hospital admission due to co-morbid physical illnesses (in particular syncope or collapse), increase in one attendance to A & E following affective disorder diagnosis, and falls before the diagnosis. Similar risk factors for fractures were noted.

Approximately 8.2% of our sample experienced a hospital admission due to either a fall or fracture. Studies on healthy populations have suggested several mechanisms which may explain these elevated risks in our sample, such as vitamin D deficiency [26, 27] and use of antidepressants [28]. Several studies also proposed a negative effect of leptin on bone mass and bone formation through a hypothalamic relay [29]. Some lifestyle factors seem to also be associated with a poor bone health, including physical inactivity [30] and alcohol consumption [31]. Previous studies have hypothesised other risk factors contributing to falls and fractures in people with depressive disorders [32], but no large-scale study has yet focused on patients with clinically diagnosed affective disorders. Our data advance the current literature by providing detailed evidence on how a multitude of demographic, medical, and psychological factors can differently affect risks of falls and fractures in people with affective disorder.

For peer review only - http://bmjopen.bmj.com/site/about/guidelines.xhtml

The association between older age and incidence of fall may be due to age-related physical conditions, as well as physiologic (e.g. loss of BMD) [33] and pathologic changes (e.g. decreased cardiovascular functions) [34]. Female gender is also a well-established risk factor for osteoporosis [35], consistent with our finding. Surprisingly, we found no relationship between deprivation and falls and fractures, although previous evidence has associated deprivation with fractures across different age groups [36, 37]. We might suppose that while deprivation is associated with poor nutrition and physical inactivity, people with affective disorders have a poorer lifestyle, compared to the general population [38], regardless of their financial status or living condition. Finally, the protective role of a non-European ethnic background is comparable with previous evidence supporting a higher fracture rate in Caucasian women compared to women from other ethnic groups [39], and a lower fall rate in older immigrants than those with an English-speaking background [40].

Our results provide new evidence on how hospitalisation due to falls or fractures can be an important burden for the NHS resources and for people with affective disorders, who are already at risk for a prolonged hospitalisation [41]. Given the long hospital stay among our sample, its burden on NHS costs in unsurprising. The total cost of fracture is estimated to reach £4.4 billion per year in the UK alone [8]. Several items from the HoNOS were also associated with increased hazard of hospitalisation due to falls or fractures (e.g. cognitive problems). Evidence on the role of these factors on hospital stay among this population is warranted for future research.

Our study reports stress-related and somatoform disorders to be associated with a decreased risk of falls and fractures, which could be due to increased fear avoidance and reduced activities due to stress-related disorders, leading to fewer falls [42]. We also found the presence of bipolar

For peer review only

affective disorder and manic episodes to be protective factors against increased fracture risk. Although there has been little evidence investigating the role of bipolar affective disorder or its characteristics (including manic symptoms) in the risk of fracture, preliminary evidence has suggested an association between the use of lithium (i.e. a primary treatment for bipolar disorder) and decreased fracture risk [43], the preservation and enhancement of bone mass [44].

Surprisingly, neither antidepressants nor antipsychotics were associated with risks of falls or fracture, despite documented associations between antipsychotic medications and reduced bone metabolism [45] and BMD [46]. Evidence on the association between antidepressants and BMD is ambiguous: while some authors reported that some antidepressants, especially SSRIs, have a negative impact on bone strength [47, 48], other findings have been null [49]. However, we did not attempt to account for specific types of antidepressants in the analysis, and previous studies indicate that tricyclic antidepressants have no adverse effect on bone health [28]. On the other hand, analgesics use was associated with increased risks for falls and fractures, consistently with previous findings [50], and possibly mediated by central nervous system effects, such as dizziness [51]. Antihypertensive drugs use was associated with a decreased fracture risk, confirming previous results [52], potentially due to their improving effect on calcium absorption [53].

Finally, extensive evidence has indicated several physical conditions as risk factors for falls or fractures in the general population [54, 55], including UTI [56] and diabetes [57]. It is known that mental health disorders (e.g. depression) increase the chance of major physical co-morbidities [58]. However, few studies have established the impact of comorbid physical conditions on risks of falls and fractures among people with affective disorders. Our study indicates heart failure, diabetes, UTI and osteoporosis to be associated with hospitalisation due

For peer review only - http://bmjopen.bmj.com/site/about/guidelines.xhtml

to falls or fractures. Hypotension and syncope or collapse were additionally associated with an increased risk of falls, and hearing loss was associated with an elevated fracture risk. Unsurprisingly, osteoporosis was strongly associated with falls and fractures, as it has been acknowledged as the most important yet potentially treatable factor for falls [54] and fractures [59] due to its effect on BMD. Moreover, osteoporosis tends to occur simultaneously with heart failure [60] due to shared risk factors such as diabetes. Unsurprisingly, syncope or collapse was also strongly associated with an increased risk of falls, as syncope is frequently mistaken for falls, to the point that the European Society of Cardiology has emphasised the need to explore syncope as an independent factor leading to falls [61]. Diabetes was another risk factor for both falls and fracture, confirming the association found in the general population [62]. Hearing loss was the only factor associated with an elevated risk of fracture in our study, an association previously confirmed in the general population [63]. This finding may be explained by the damaging impact of hearing loss on orientation skills and physical activity [64]. Finally, our study confirmed the association between history of falls and increased risk of future falls, previously reported in the general population [34, 65] and in people with depression [66]. Moreover, our data confirmed how a prior fracture represents a 50-100% higher risk for experiencing future fracture, as reported in the general population [67].

The main strengths of our study are: i) analyses controlling for various confounders; and ii) a large sample including people with clinically diagnosed affective disorders. However, there are important limitations. Firstly, we did not stratify according to different affective disorder subtypes which could report different risk factors; similarly, we analysed psychotropic medication categories but did not investigate specific agents or sub-categories. Second, we had no information on the types of fractures reported at admission and on factors (e.g. balance) that are influenced by neuropathological lesions in people with depression [68]. Additionally, the

For peer review only - http://bmjopen.bmj.com/site/about/guidelines.xhtml

majority of studies investigating falls and fractures rely upon self-report data whilst our study relied on ICD hospitalisation codes increasing reliability. However, the causes of fractures and the types of falls could not be explored in the current study, although we investigate factors associated with falls across the whole sample from the rich mental health record database. Future research should attempt to disentangle of the potential risk factors/causes of falls and fractures research in affective disorders. A limitation of health administrative data is some inconsistency or limited data on the granular detail. Third, lifestyle factors, such as alcohol consumption [31, 38], could not be explored. Fourth, our study only ascertained information on physical co-morbidities from hospitalisation records and could have overlooked patients with mild osteoporosis. Additionally, physical co-morbidities were ascertained within six months of diagnosis, and patients who were hospitalised for a fall or fracture within these six months would be more likely to have their osteoporosis ascertained. Fifth, falls and fractures are closely related and often co-occur [69]. In this study, although falls and fractures were identified from ICD hospitalisation codes, falls and fractures categories were not mutually exclusive, as a patient could be counted in both the fall and fracture cohort if he/she had a fall and a fracture in the same event. Moreover, factors such as inaccurate recording and fine distinction between ICD hospitalisation codes for falls and fractures, could also potentially hinder our attempt to differentiate the two concepts. Thus, further work is needed to elucidate the potential overlap and distinct relationship among falls and fractures in people with affective disorders. Finally, since medication records were ascertained within one year of diagnosis, we cannot assess whether medications were prescribed as a result or as a cause of falls for those who had a fall or fracture within six months of their diagnosis.

For peer review only

For peer review only - http://bmjopen.bmj.com/site/about/guidelines.xhtml

Conclusion

Our study reports that over a mean 5 years' follow-up, approximately 8% of a large cohort with affective disorders was hospitalised due to either a fall or fracture. Factors such as older age, certain medications and having a history of fall or fracture are significant predictors, broadly comparable to risk factors established in the general population. Heavy hospital burden following a fall or fracture was also described.

Our current study provides important implications for future research and clinical practice. Future research should take into account the limitations listed above and potentially explore the mechanisms through which the factors identified in this study (e.g., physical conditions such as UTI, or medication use) may impact risks for falls and fractures in people with affective disorders. The associations between certain factors (e.g., psychotropic medication use) and risk for falls and fractures were not confirmed in the current study, future studies are encouraged to replicate our findings and explore these factors further.

In terms of implications for future clinical practice, given the lack of osteoporosis screening for people with mental health problems in current clinical settings [70], BMD checks and osteoporosis screenings should be a routine for people with affective disorders, especially if older and with established co-morbid chronic illnesses. Fall assessments (e.g., the Fracture Risk Assessment Tool) should be routinely conducted for people with affective disorders, since approximately 70% of low-energy fractures are due to falls [71]. Fall/fracture prevention programmes should also be offered due to an average 14% reduction in falls risk in the general population following fall prevention programmes [72]. For example, the combination of cognitive behavioural therapy and exercise have been previously found to be effective in improving depressive symptoms [73] and self-efficacy [74] in the general population. The effectiveness of these prevention programmes should also be examined in people with affective disorders and subsequently provided if their benefits are confirmed.

For peer review only - http://bmjopen.bmj.com/site/about/guidelines.xhtml

Acknowledgments

Author's contributions

BS acquired funding for the study. GP conducted the analysis with support from all co-authors. RM drafted introduction and discussion sections, ER drafted results section and all authors (BS, GP, DV, AK, RS, CM) provided critical revisions and approved the final version. The PPI group helped conceptualize and co-develop the study, as well as contributed to the funding applications.

Financial support

BS is supported by a Clinical Lectureship (ICA-CL-2017-03-001) jointly funded by Health Education England (HEE) and the National Institute for Health Research (NIHR). BS and RS are part funded by the NIHR Biomedical Research Centre at South London and Maudsley NHS Foundation Trust. RM is part funded by the HDR-UK London award to King's College London. The views expressed are those of the author(s) and not necessarily those of the (partner organisation), the NHS, the NIHR or the Department of Health and Social Care.

Ethics approval

This study has full approval for secondary analysis of CRIS (Oxford Research Ethnic Committee C, reference 18/SC/0372).

Conflicts of interest

All authors declare no conflicts of interest.

Data availability statement

For peer review only

Data are available on reasonable request. For questions regarding the study, please contact RM at ruimin.1.ma@kcl.ac.uk

For peer review only

References

[1] Hernlund E, Svedbom A, Ivergård M, Compston J, Cooper C, Stenmark J, McCloskey EV, Jönsson B, Kanis JA. Osteoporosis in the European Union: Medical management, epidemiology and economic burden: A report prepared in collaboration with the International Osteoporosis Foundation (IOF) and the European Federation of Pharmaceutical Industry Associations (EFPIA). *Archives of Osteoporosis*. 2013 https://doi.org/10.1007/s11657-013-0136-1

[2] Panel on Prevention of Falls in Older Persons, American Geriatric Society and British Geriatric Society. Summary of the updated American Geiratric Society/British Geiratric Society clincial practice guideline for prevention of falls in older persons. Journal of American Geiratric Society, 2011; 59(1): 148-157

[3] Sinaki, M. Falls, fractures, and hip pads. *Current osteoporosis reports,* 2004; 2(4): 131-137.

[4] Dionyssiotis Y. Analysing the problem of falls among older people. International Journal of General Medicine, 2012; 5: 805-813.

[5] Deandrea S, Lucenteforte E, Bravi F, Foschi R, La Vecchia C, Negri E. Risk factors for falls in community-dwelling older people: A systematic review and meta-analysis. In *Epidemiology* 2010: pp. 658–668. https://doi.org/10.1097/EDE.0b013e3181e89905

[6] NICE. Falls in older people: assessing risk and prevention. 2013. Available at https://www.nice.org.uk/guidance/cg161. Accessed 13 March 2020

[7] Sanguineti VA, Wild JR, Joseph B, Fain MJ. Management of common fractures in older adults. In Michel, J-P., Beattie, B. L., Martin, F. C., & Walston, J (eds). Oxford Textbookof Geriatric Medictine (3ed).2017. Oxford University Press. DOI: 10.1093/med/9780198701590.003.0070

[8] Public Health England. Falls: applying all our health. 2020. Available at

For peer review only - http://bmjopen.bmj.com/site/about/guidelines.xhtml

https://www.gov.uk/government/publications/falls-applying-all-our-health/falls-applying-all-our-health. Accessed 13 March 2020

[9] Johnell O, Kanis JA. An estimate of the worldwide prevalence and disability associated with osteoporotic fractures. Osteoporos. Int. 2006; 17 (12): 1726–1733

[10] Correll CU, Detraux J, De Lepeleire J, De Hert M. Effects of antipsychotics, antidepressants and mood stabilizers on risk for physical diseases in people with schizophrenia, depression and bipolar disorder. World Psychiatry, 2015; 14, 119–136. https://doi.org/10.1002/wps.20204

[11] Shi TT, Min M, Zhang Y, Sun CY, Liang MM, Sun YH. Depression and risk of hip fracture: a systematic review and meta-analysis of cohort studies. In Osteoporosis International, 2019; 30(6): 1157–1165. https://doi.org/10.1007/s00198-019-04951-6

[12] Chandrasekaran V, Brennan-Olsen SL, Stuart AL, Pasco JA, Berk M, Hodge JM, Williams LJ. Bipolar disorder and bone health: A systematic review. Journal of Affective Disorders, 2019; 249: 262–269. https://doi.org/10.1016/j.jad.2019.02.013

[13] Hong-Jhe C, Chin-Yuan, K, Ming-Shium T, Fu-Wei W, Ru-Yih C, Kuang-Chieh H, Hsiang-Ju P, Ming-Yueh C, Pan-Ming C, Chih-Chuan P. The incidence and risk of osteoporosis in patients with anxiety disorder: A Population-based retrospective cohort study. *Medicine*, 2016; *95*(38): e4912. https://doi.org/10.1097/MD.0000000000004912

[14] Wu Q, Liu B, Tonmoy S. Depression and risk of fracture and bone loss: an updated meta-analysis of prospective studies. *Osteoporosis International*, 2018; *29*: 1303–1312. https://doi.org/10.1007/s00198-018-4420-1

[15] Stubbs B, Stubbs J, Gnanaraj SD, Soundy A. Falls in older adults with major depressive disorder (MDD): a systematic review and exploratory meta-analysis of prospective studies. Int Psychogeriatr, 2016; 28(1): 23-29. doi: 10.1017/S104161021500126X.

[16] Fernandes AC, Cloete D, Broadbent MTM, Hayes RD, Chang CK., Jackson RG, et al.

For peer review only

Development and evaluation of a de-identification procedure for a case register sourced from mental health electronic records. *BMC Medical Informatics and Decision Making*, 2013; *13*: 71. https://doi.org/10.1186/1472-6947-13-71

[17] Perera G, Broadbent M, Callard F, Chang CK, Downs J, Dutta R, et al. Cohort profile of the South London and Maudsley NHS Foundation Trust Biomedical Research Centre (SLaM BRC) Case Register: Current status and recent enhancement of an Electronic Mental Health Record-derived data resource. *BMJ Open*, 2016; *6*(3): e008721. https://doi.org/10.1136/bmjopen-2015-008721

[18] Sultana J, Chang CK, Hayes RD, Broadbent M, Stewart R, Corbett A, Ballard C. Associations between risk of mortality and atypical antipsychotic use in vascular dementia: A clinical cohort study. *International Journal of Geriatric Psychiatry*, 2014; *29*(12): 1249–1254. https://doi.org/10.1002/gps.4101

[19] Ward G, Perera G, Stewart R. Predictors of mortality for people aged over 65 years receiving mental health care for delirium in a South London Mental Health Trust, UK: A retrospective survival analysis. *International Journal of Geriatric Psychiatry*, 2015; *30*(6): 639–646. https://doi.org/10.1002/gps.4195

[20] Mueller C, Perera G, Hayes RD, Shetty H, Stewart R. Associations of acetylcholinesterase inhibitor treatment with reduced mortality in Alzheimer's disease: A retrospective survival analysis. *Age and Ageing*, 2018; *47*: 88–94. https://doi.org/10.1093/ageing/afx098

[21] Stubbs B, Mueller C, Gaughran F, Lally J, Vancampfort D, Lamb SE, et al. Predictors of falls and fractures leading to hospitalisation in people with schizophrenia spectrum disorder: a large representative cohort study. Schizophrenia Research, 2018; 201: 70-78

[22] Das-Munshi J, Chang C-K, Dutta R et al. Ethnicity and excess mortality in severe mental illness: a cohort study. The Lancet 2017; 4(5): 389-399. Doi:

For peer review only - http://bmjopen.bmj.com/site/about/guidelines.xhtml

https://doi.org/10.1016/S2215-0366(17)30097-4

[23] Richardson EA, Mitchell RJ, Shortt NK et al. Evidence-based selection of environmental factors and datasets for measuring multiple environmental deprivation in epidemiological research. Environ Health, 2009; 8: S18. DOI: https://doi.org/10.1186/1476-069X-8-S1-S18

[24] Wing JK, Beevor AS, Curtis RH, Park SGB, Hadden J, Burns, A. Health of the Nation Outcomes Scales (HoNOS): Research and development. The British Journal of Psychaitry, 1998; 172(1):11-18. DOI: https://doi.org/10.1192/bjp.172.1.11

[25] Burns A, Beevor A, Lelliott P, Wing J, Blakey A, Orrell M, et al. Health of the Nation Outcome Scales for Elderly People (HoNOS 65+): Glossary for HoNOS 65+ score sheet. Cambridge University Press, 1999; 174(5): 435-438. DOI: https://doi.org/10.1192/bjp.174.5.435

[26] Kahl KG, Greggersen W, Rudolf S, Stoeckelhuber BM, Bergmann-Koester CU, Dibbelt L, Schweiger U. Bone mineral density, bone turnover, and osteoprotegerin in depressed women with and without borderline personality disorder. *Psychosomatic Medicine*, 2006; *68*: 669–674. https://doi.org/10.1097/01.psy.0000237858.76880.3d

[27] Kahl KG, Rudolf S, Dibbelt L, Stoeckelhuber BM, Gehl HB, Hohagen F, Schweiger U. Decreased osteoprotegerin and increased bone turnover in young female patients with major depressive disorder and a lifetime history of anorexia nervosa. *Osteoporosis International*, 2005; *16*: 424–429. https://doi.org/10.1007/s00198-004-1711-5

[28] Diem SJ, Blackwell TL, Stone KL, Yaffe K, Haney EM, Bliziotes MM, Ensrud, KE. Use of antidepressants and rates of hip bone loss in older women: The study of osteoporotic fractures. *Archives of Internal Medicine*, 2007; *167*: 1240–1245. https://doi.org/10.1001/archinte.167.12.1240

[29] Trivedi R, Goswami R, Chattopadhyay N. Invesgitational anabolic therapies for

For peer review only

osteoporosis. Expert Opin Investig Drugs, 2010; 19(8): 995-1005

[30] Wong SYS, Lau EMC, Lynn H, Leung PC, Woo J, Cummings SR, Orwoll E. Depression and bone mineral density: Is there a relationship in elderly Asian men? Results from Mr. Os (Hong Kong). Osteoporosis International, 2005; 16: 610–615. https://doi.org/10.1007/s00198-004-1730-2

[31] Mussolino ME. Depression and hip fracture risk: The NHANES I epidemiologic follow-up study. Public Health Reports, 2005; 120: 71–75. https://doi.org/10.1177/003335490512000112

[32] Cizza G. Major depressive disorder is a risk factor for low bone mass, central obesity, and other medical conditions. Dialogues in Clinical Neuroscience, 2011; 13(1): 73–87.

[33] Wu Q, Magnus JH, Liu J, Bencaz AF, Hentz JG. (2009). Depression and low bone mineral density: A meta-analysis of epidemiologic studies. Osteoporosis International, 2009; 20(8): 1309–1320. https://doi.org/10.1007/s00198-009-0918-x

[34] Ambrose AF, Paul G, Hausdorff JM. (2013). Risk factors for falls among older adults: A review of the literature. Maturitas, 2013; 75(1): 51–61. https://doi.org/10.1016/j.maturitas.2013.02.009

[35] Williams J, Kool B, Robinson E,Ameratunga S. Longer term health of young and middle-aged adults following unintentional falls at home resulting in hospitalisation. *Injury*, 2012; *43*(1): 103–108. https://doi.org/10.1016/j.injury.2011.03.050

[36] Court-Brown CM, Aitken SA, Duckworth AD, Clement ND, McQueen MM. The relationship between social deprivation and the incidence of adult fractures. *Journal of Bone and Joint Surgery - Series A*, 2013; *95*(6): e321–e327. https://doi.org/10.2106/JBJS.K.00631

[37] Menon MRG, Walker JL, Court-Brown CM. The epidemiology of fractures in adolescents with reference to social deprivation. *Journal of Bone and Joint Surgery -*

For peer review only

Series B, 2008; *90*(11): 1482–1486. https://doi.org/10.1302/0301-620X.90B11.21163

[38] Eskandari F, Martinez PE, Torvik S, Phillips TM, Sternberg EM, Mistry S, et al. Low bone mass in premenopausal women with depression. *Archives of Internal Medicine*, 2007; *167*: 2329–2336. https://doi.org/10.1001/archinte.167.21.2329

[39] Cauley JA, Chalhoub D, Kassem AM, Fuleihan GEH. Geographic and ethnic disparities in osteoporotic fractures. *Nature Reviews Endocrinology,* 2014; 10(6): 338–351. https://doi.org/10.1038/nrendo.2014.51

[40] Bradley C, Harrison JE. (2007). Fall-related hospitalisations among older people: Sociocultural and regional aspects. *AIHW*. Available at: https://www.aihw.gov.au/getmedia/17a9a4b4-32a1-4481-8a42-b67ec21db92f/10447.pdf.aspx?inline=true, accessed on 28th March 2020

[41] Wolff J, McCrone P, Patel A, Kaier K, Normann C. Predictors of length of stay in psychiatry: Analyses of electronic medical records. *BMC Psychiatry*, 2015;*15*(1): 238. https://doi.org/10.1186/s12888-015-0623-6

[42] Stubbs B, Patchay B, Soundy A, Schofield P. The avoidance of activities due to fear of falling contribute to sedentary behavior among community-dwelling older adults with chronic musculoskeletal pain: a multisite observational study. Pain Med, 2014; 15(11): 1861-1871. doi: 10.1111/pme.12570.

[43] Vestergaard P. Skeletal effects of central nervous system active drugs: anxiolytics, sedatives, antidepressants, lithum and neuroletpics. Curr Drug Saf, 2008; 3: 185-189. DOI : 10.2174/157488608785699432

[44] Zamani A, Omrani GR, Nasab MM. Lithium's effect on bone mineral density. Bone, 2009; 44: 331-334. doi: 10.1016/j.bone.2008.10.001.

[45] Klibanski A, Biller BMK, Rosenthal DI, Schoenfeld DA, Saxe V. Effects of prolactin and estrogen deficiency in amenorrheic bone loss. *Journal of Clinical Endocrinology*

For peer review only

and Metabolism, 1988; *67*: 124–130. https://doi.org/10.1210/jcem-67-1-124

[46] Crews MPK, Howes OD. Is antipsychotic treatment linked to low bone mineral density and osteoporosis? A review of the evidence and the clinical implications. In *Human Psychopharmacology* 2012; 27(1): 15–23. https://doi.org/10.1002/hup.1265

[47] Coupland C, Dhiman P, Morriss R, Arthur A, Barton G, Hippisley-Cox J. Antidepressant use and risk of adverse outcomes in older people: Population based cohort study. *BMJ (Online)*, 2011; *343*: 4551. https://doi.org/10.1136/bmj.d4551

[48] Rauma PH, Honkanen RJ, Williams LJ, Tuppurainen MT, Kröger HP, Koivumaa-Honkanen H. Effects of antidepressants on postmenopausal bone loss - A 5-year longitudinal study from the OSTPRE cohort. *Bone*, 2016; *89*: 25–31. https://doi.org/10.1016/j.bone.2016.05.003

[49] Ruths S, Bakken MS, Ranhoff AH, Hunskaar S, Engesæter LB, Engeland A. Risk of hip fracture among older people using antihypertensive drugs: A nationwide cohort study. *BMC Geriatrics*, 2015; *15*: 153. https://doi.org/10.1186/s12877-015-0154-5

[50] Rossini M, Viapiana O, Adami S, Idolazzi L, Buda S, Veronesi C, Degli Esposti L, Gatti D. Medication use before and after hip fracture: A population-based cohort and case-control study. *Drugs and Aging*, 2014; *31*: 547–553. https://doi.org/10.1007/s40266-014-0184-2

[51] Vestergaard P, Rejnmark L, Mosekilde L. Fracture risk associated with the use of morphine and opiates. *Journal of Internal Medicine*, 2006; *260*(1): 76–87. https://doi.org/10.1111/j.1365-2796.2006.01667.x

[52] Solomon DH, Mogun H, Garneau K, Fischer MA. Risk of fractures in older adults using antihypertensive medications. *Journal of Bone and Mineral Research*, 2011; *26*(7): 1561–1567. https://doi.org/10.1002/jbmr.356

[53] Pérez-Castrillón JL, Silva J, Justo I, Sanz A, Martín-Luquero M, Igea R, et al. Effect of

For peer review only

For peer review only - http://bmjopen.bmj.com/site/about/guidelines.xhtml

quinapril, quinapril-hydrochlorothiazide, and enalapril on the bone mass of hypertensive subjects: Relationship with angiotensin converting enzyme polymorphisms. *American Journal of Hypertension*, 2003; *16*: 453–459. https://doi.org/10.1016/S0895-7061(03)00845-8

[54] Choi EJI, Kim SA, Kim NRI, Rhee JA, Yun YW, Shin MH. Risk factors for falls in older Korean adults: the 2011 Community Health Survey. *Journal of Korean Medical Science*, 2014; *29*(11): 1482–1487. https://doi.org/10.3346/jkms.2014.29.11.1482

[55] Menant JC, Wong AKW, Trollor JN, Close JCT, Lord SR. Depressive Symptoms and Orthostatic Hypotension Are Risk Factors for Unexplained Falls in Community-Living Older People. *Journal of the American Geriatrics Society*, 2016; *64*: 1073–1078. https://doi.org/10.1111/jgs.14104

[56] Soliman Y, Meyer R, Baum N. (2016). Falls in the Elderly Secondary to Urinary Symptoms. *Reviews in Urology*, 2016; *18*(1): 28–32.

[57] Wang J, You W, Jing Z, Wang R, Fu Z, Wang Y. Increased risk of vertebral fracture in patients with diabetes: a meta-analysis of cohort studies. In *International Orthopaedics* 2016; 40(6): 1299–1307. https://doi.org/10.1007/s00264-016-3146-y

[58] Ojike N, Sowers JR, Seixas A, Ravenell J, Rodriguez-Figueroa G, Awadallah M, et al. Psychological distress and hypertension: results from the national health interview survey for 2004-2013. *CardioRenal Medicine*, 2016; *6*(3): 198–208. https://doi.org/10.1159/000443933

[59] Friedman SM, Mendelson DA. Epidemiology of fragility fractures. *Clinics in Geriatric Medicine* 2014; 30(2): 175–181. https://doi.org/10.1016/j.cger.2014.01.001

[60] Carbone LD, Bková P, Fink HA, Lee JS, Chen Z, Ahmed A, Parashar S, Robbins JR. Hip fractures and heart failure: Findings from the Cardiovascular Health Study. *European Heart Journal*, 2010; *31*: 77–84. https://doi.org/10.1093/eurheartj/ehp483

For peer review only

For peer review only - http://bmjopen.bmj.com/site/about/guidelines.xhtml

[61] Moya A, Sutton R, Ammirati F, Blanc JJ, Brignole M, Dahm JB, et al. Guidelines for the diagnosis and management of syncope (version 2009). *European Heart Journal 2009;* 30(21): e2631–e2671. https://doi.org/10.1093/eurheartj/ehp298

[62] López-Ibarra PJ, Pastor MMC, Escobar-Jiménez F, Pardo MDS, GonzĺEz AG, De Dios Luna J, et al. Bone mineral density at time of clinical diagnosis of adult-onset type 1 diabetes mellitus. *Endocrine Practice*, 2001; *7*: 346–351. https://doi.org/10.4158/EP.7.5.346

[63] Kim S-Y, Lee J-K, Sim S, Choi HG. Hearing impairement increases the risk of distal radius, hip and spine fractures: a longitudinal follow-up study using a national sample cohort. PLoS One, 2018; 13(2): e0192820. doi: 10.1371/journal.pone.0192820

[64] Keller BK, Morton JL, Thomas VS, Potter JF. The effect of hearing and visual impairments on functional status. J Am Geriatr Soc, 1999; 47(11): 1319-1325. https://doi.org/10.1111/j.1532-5415.1999.tb07432.x

[65] American Geriatrics Society, British Geriatrics Society, and A. A. of, & Orthopaedic Surgeons. Guideline for the Prevention of Falls in Older Persons and American Academy of Orthn of opaedic Surgeons Panel on Falls Prevention. *American Geriatrics Society*, 2001; *49*(5): 664–672.

[66] Briggs R, Kennelly SP, Kenny RA. Does baseline depression increase the risk of unexplained and accidental falls in a cohort of community-dwelling older people? Data from The Irish Longitudinal Study on Ageing (TILDA). *International Journal of Geriatric Psychiatry*, 2018; *33*(2): e205–e211. https://doi.org/10.1002/gps.4770

[67] Van Den Berg M, Verdijk NA, Leusink GL, Wijnands-Van Gent CJM., Romeijnders AC, Pop VJM, et al. Depression after low-energy fracture in older women predicts future falls: A prospective observational study. *BMC Geriatrics*, 2011; *11*: 73. https://doi.org/10.1186/1471-2318-11-73

For peer review only - http://bmjopen.bmj.com/site/about/guidelines.xhtml

[68] Lee SC, Hu LY, Huang MW, Shen CC, Huang WL, Lu T, Hsu CL, Pan CC. Risk of vertebral fracture in patients diagnosed with a depressive disorder: A nationwide population-based cohort study. *Clinics*, 2017; *72*(1): 44–50. https://doi.org/10.6061/clinics/2017(01)08

[69] Berry SD & Miller R. Falls: epidemiology, pathphysiology and relationship to fracture. *Curr Osteroporos rep*, 2008; 6(4):149-154

[70] Gleason LJ, Menzies IB, Mendelson DA, Kates SL, Friedman SM. Diagnosis and Treatment of Osteoporosis in High-Risk Patients Prior to Hip Fracture. *Geriatric Orthopaedic Surgery & Rehabilitation*, 2012; *3*(2): 79–83. https://doi.org/10.1177/2151458512454878

[71] Appleby PN, Allen NE, Roddam AW, Key TJ. Physical activity and fracture risk: A prospective study of 1898 incident fractures among 34 696 British men and women. *Journal of Bone and Mineral Metabolism*, 2008; *26*: 191–198. https://doi.org/10.1007/s00774-007-0806-4

[72] Choi M, Hector M. Effectiveness of intervention programs in preventing falls: A systematic review of recent 10 years and meta-analysis. *Journal of the American Medical Directors Association*, 2012; *13*(2): e13–e21. https://doi.org/10.1016/j.jamda.2011.04.022

[73] Krogh J, Nordentoft M, Sterne JAC, Lawlor DA. The effect of exercise in clinically depressed adults: Systematic review and meta-analysis of randomized controlled trials. *Journal of Clinical Psychiatry*, 2011; *72*: 529–538. https://doi.org/10.4088/JCP.08r04913blu

[74] Zijlstra GAR, Van Haastregt JCM, Ambergen T, Van Rossum E, Van Eijk JTM, Tennstedt SL, Kempen GIJM. Effects of a multicomponent cognitive behavioral group intervention on fear of falling and activity avoidance in community-dwelling older

For peer review only - http://bmjopen.bmj.com/site/about/guidelines.xhtml

adults: Results of a randomized controlled trial. *Journal of the American Geriatrics Society*, 2009; *57*(11): 2020–2028. https://doi.org/10.1111/j.1532-5415.2009.02489.x

For peer review only

Table 1: Characteristics of those Mood [affective] disorders patients who were admitted to hospital with a fall/fracture after a diagnosis of Mood [affective] disorders (F30* to F34*).

	Presence of falls		Presence of fractures	
**Characteristics of sample**	**No (n= 34,270)**	**Yes (n= 1,831)**	**No (n= 33,908)**	**Yes (n= 2,193)**
**Age at the time of Mood [affective] disorders diagnosis**				
18- 34	12685 (37.0)	205 (11.2)	12572 (37.1)	318 (14.5)
35- 49	11409 (33.3)	336 (18.4)	11346 (33.5)	399 (18.2)
50- 64	5829 (17.0)	362 (19.8)	5751 (17.0)	440 (20.1)
65- 79	2797 (8.2)	485 (26.5)	2728 (8.0)	554 (25.3)
80 & over	1550 (4.5)	443 (24.2)	1511 (4.5)	482 (22.0)
**Gender**				
Female	20627 (60.2)	1107 (60.5)	20348 (60.0)	1386 (63.2)
Male	13638 (39.8)	724 (39.5)	13555 (40.0)	807 (36.8)
**Ethnicity**				
White	20772 (60.6)	1519 (83.0)	20536 (60.6)	1755 (80.0)
Non-white	12450 (36.3)	288 (15.7)	12333 (36.4)	405 (18.5)
**Marital status**				
Cohabiting	7957 (23.2)	416 (22.7)	7867 (23.2)	506 (23.1)
Non-cohabiting	23028 (67.2)	1345 (73.5)	22776 (67.2)	1597 (72.8)
**Index of multiple deprivation: IMD 2015 (SD)**	27.97 (11.31)	27.90 (11.53)	27.97 (11.30)	27.97 (11.52)
Least deprived quintile	6657 (19.4)	388 (21.2)	6593 (19.4)	452 (20.6)
2nd most deprived quintile	6682 (19.5)	378 (20.6)	6643 (19.6)	417 (19.0)
3rd most deprived quintile	6706 (19.6)	354 (19.3)	6637 (19.6)	423 (19.3)
Most deprived quintile	6708 (19.6)	346 (18.9)	6612 (19.5)	442 (20.2)
**Medication prescription (within 6 months before or after Mood [affective] disorders diagnosis)**				

For peer review only

Anticholinergics	17714 (51.7)	1089 (59.5)	17489 (51.6)	1314 (59.9)
Antihypertensives	2561 (7.5)	386 (21.1)	2553 (7.5)	394 (18.0)
Antidepressants	19351 (56.5)	1168 (63.8)	19113 (56.4)	1406 (64.1)
Antipsychotics	8777 (25.6)	469 (25.6)	8694 (25.6)	552 (25.2)
Anxiolytics and Hypnotics	9443 (27.6)	558 (30.5)	9349 (27.6)	652 (29.7)
Analgesics	3083 (9.0)	405 (22.1)	3059 (9.0)	429 (19.6)
**Number of medications received (within 6 months before or after Mood [affective] disorders diagnosis)**				
0	10207 (29.8)	394 (21.5)	10126 (29.9)	475 (21.7)
1	5330 (15.6)	259 (14.1)	5275 (15.6)	314 (14.3)
2	7062 (20.6)	360 (19.7)	6961 (20.5)	461 (21.0)
3	6599 (19.3)	361 (19.7)	6503 (19.2)	457 (20.8)
4	3858 (11.3)	304 (16.6)	3839 (11.3)	323 (14.7)
5	1038 (3.0)	121 (6.6)	1029 (3.0)	130 (5.9)
6	176 (0.5)	32 (1.7)	175 (0.5)	33 (1.5)
**Other psychiatric conditions (within six months before or after Mood [affective] disorders diagnosis)**				
F00- F03 (Dementia)	754 (2.2)	207 (11.3)	749 (2.2)	212 (9.7)
F 20- F29 (Schizophrenia spectrum disorder)	2701 (7.9)	119 (6.5)	2676 (7.9)	144 (6.6)
F30- F31 (Bipolar affective disorder)	5489 (16.0)	222 (12.1)	5475 (16.1)	236 (10.8)
F50 (Eating Disorders)	447 (1.3)	13 (0.7)	441 (1.3)	19 (0.9)
F40- F48 (Neurotic, stress-related and somatoform disorders)	3652 (10.7)	147 (8.0)	3601 (10.6)	198 (9.0)
F 60 (Disorders of adult personality and behaviour)	1436 (4.2)	77 (4.2)	1428 (4.2)	85 (3.9)
**Problem HoNOS (score 2 or over) (within six months before or after Mood [affective] disorders diagnosis)**				
Agitated Behaviour	3571 (10.4)	234 (12.8)	3547 (10.5)	258 (11.8)
Self-Injury	3586 (10.5)	186 (10.2)	3545 (10.5)	227 (10.4)
Problem Drinking Drugs	2957 (8.6)	204 (11.1)	2932 (8.6)	229 (10.4)

For peer review only - http://bmjopen.bmj.com/site/about/guidelines.xhtml

Cognitive Problems	2821 (8.2)	349 (19.1)	2792 (8.2)	378 (17.2)
Physical Illness	6514 (19.0)	770 (42.1)	6355 (18.7)	929 (42.4)
Hallucinations	3198 (9.3)	175 (9.6)	3176 (9.4)	197 (9.0)
Depressed Mood	14582 (42.6)	766 (41.8)	14390 (42.4)	958 (43.7)
Relationship Problems	7644 (22.3)	342 (18.7)	7560 (22.3)	426 (19.4)
Daily Living Problems	5854 (17.1)	576 (31.5)	5723 (16.9)	707 (32.2)
Living Conditions Problems Score	3431 (10)	190 (10.4)	3404 (10.0)	217 (9.9)
Occupational Problems	5574 (16.3)	392 (21.4)	5513 (16.3)	453 (20.7)
Mean overall HoNoS score (SD)	10.67 (5.63)	11.64 (5.53)	10.64 (5.63)	11.91 (5.46)
Number with missing HoNoS	12545 (36.6)	523 (28.6)	12411 (36.6)	657 (30.0)
**Hospital admissions (within six months before or after Mood [affective] disorders diagnosis)**				
Ischaemia +CHD+ IHD	1128 (3.3)	210 (11.5)	1089 (3.2)	249 (11.4)
Arrhythmia + AF	1003 (2.9)	197 (10.8)	967 (2.9)	233 (10.6)
Heart failure	437 (1.3)	88 (4.8)	417 (1.2)	108 (4.9)
Diabetes	1516 (4.4)	264 (14.4)	1488 (4.4)	292 (13.3)
Hypotension	435 (1.3)	104 (5.7)	424 (1.3)	115 (5.2)
Hypercholesterolemia	1097 (3.2)	221 (12.1)	1090 (3.2)	228 (10.4)
Hypertension	2837 (8.3)	546 (29.8)	2752 (8.1)	631 (28.8)
Urinary tract infections (UTI)	1366 (4.0)	324 (17.7)	1297 (3.8)	393 (17.9)
Osteoporosis	409 (1.2)	121 (6.6)	183 (0.5)	347 (15.8)
Visual Disturbance and Blindness	241 (0.7)	53 (2.9)	246 (0.7)	48 (2.2)
Hearing Loss	189 (0.6)	41 (2.2)	174 (0.5)	56 (2.6)
Syncope or Collapse	1990 (5.8)	444 (24.2)	2022 (6.0)	412 (18.8)
Parkinson's Disease	116 (0.3)	45 (2.5)	128 (0.4)	33 (1.5)
Falls before diagnosis	750 (2.2)	267 (14.6)	749 (2.2)	268 (12.2)
Fractures before diagnosis	1075 (3.1)	231 (12.6)	860 (2.5)	446 (20.3)

For peer review only - http://bmjopen.bmj.com/site/about/guidelines.xhtml

mean number of attendances to A&E following Mood [affective] disorders diagnosis (SD)	3.94 (9.98)	16.64 (28.88)	4.00 (9.62)	13.67 (29.35)

For peer review only

Table 2: Univariate Cox proportional hazard model (95% CI) showing factors affecting time to first fall/fracture hospital admission since diagnosis of Mood [affective] disorders

	Outcome falls		Outcome fractures	
**Characteristics**	**HR (95% CI)**	**P value**	**HR (95% CI)**	**P value**
**Age at the time of Mood [affective] disorders diagnosis**				
18- 34	Ref.		Ref.	
35- 49	1.74 (1.46, 2.07)	**<0.001**	1.33 (1.15, 1.54)	**<0.001**
50- 64	3.88 (3.27, 4.61)	**<0.001**	3.03 (2.62, 3.50)	**<0.001**
65- 79	12.41 (10.54, 14.62)	**<0.001**	9.11 (7.94, 10.46)	**<0.001**
80 & over	26.94 (22.8, 31.83)	**<0.001**	18.46 (16.00, 21.29)	**<0.001**
Female gender	0.99 (0.90, 1.09)	0.87	1.12 (1.03, 1.22)	**0.01**
Non-European ethnicity	0.32 (0.28, 0.37)	**<0.001**	0.39 (0.35, 0.44)	**<0.001**
Non-cohabiting marital status	1.08 (0.97, 1.21)	0.17	1.05 (0.95, 1.16)	0.31
**Deprivation quintile (IMD 2015)**				
Least deprived quintile	Ref.		Ref.	
2nd least deprived quintile	0.83 (0.71, 0.96)	**0.01**	0.89 (0.78, 0.99)	**0.04**
3rd most deprived quintile	0.88 (0.76, 1.02)	**0.09**	0.90 (0.79, 1.03)	0.13
2nd most deprived quintile	0.94 (0.82, 1.09)	0.43	0.89 (0.78, 1.00)	**0.05**
Most deprived quintile	0.86 (0.74, 0.99)	**0.04**	0.94 (0.82, 1.07)	0.35
**Medication prescribed (within one year before or after Mood [affective] disorders diagnosis)**				
Anticholinergics received	1.42 (1.3, 1.56)	**<0.001**	1.45 (1.33, 1.57)	**<0.001**
Antihypertensive received	3.69 (3.3, 4.13)	**<0.001**	2.98 (2.67, 3.33)	**<0.001**
Antidepressants received	1.41 (1.29, 1.56)	**<0.001**	1.43 (1.31, 1.56)	**<0.001**

For peer review only

Antipsychotics received	0.95 (0.86, 1.06)	0.35	0.93 (0.84, 1.02)	0.12
Anxiolytics and Hypnotics received	1.11 (1.00, 1.22)	**0.05**	1.06 (0.97, 1.17)	0.18
Analgesics received	2.97 (2.66, 3.32)	**<0.001**	2.52 (2.27, 2.80)	**<0.001**
Increase in one type of polypharmacy	1.21 (1.18, 1.25)	**<0.001**	1.18 (1.15, 1.21)	**<0.001**
**Presence of other psychiatric conditions (within one year before or after Mood [affective] disorders diagnosis)**				
F00- F03 (Dementia)	6.90 (5.97, 7.98)	**<0.001**	5.65 (4.90, 6.51)	**<0.001**
F20- F29 (Schizophrenia spectrum disorder)	0.75 (0.62, 0.91)	**<0.001**	0.76 (0.64, 0.90)	**0.01**
F30- F31 (Bipolar affective disorder)	0.71 (0.62, 0.82)	**<0.001**	0.61 (0.54, 0.71)	**<0.001**
F40- F48 (Neurotic, stress-related and somatoform disorders)	0.76 (0.64, 0.90)	**<0.001**	0.86 (0.75, 0.99)	**0.05**
F50 (Eating Disorders)	0.53 (0.31, 0.91)	**0.02**	0.64 (0.41, 1.01)	0.06
F 60 (Disorders of adult personality and behaviour)	0.95 (0.76, 1.20)	0.68	0.87 (0.7, 1.09)	0.22
**Problem in HoNoS (scored 2 or more)**				
Agitated Behaviour	1.11 (0.97, 1.28)	0.14	1.03 (0.90, 1.17)	0.71
Self-Injury	0.84 (0.72, 0.98)	**0.02**	0.87 (0.76, 1.01)	0.06
Problem Drinking Drugs	1.11 (0.96, 1.29)	0.17	1.05 (0.91, 1.21)	0.48
Cognitive Problems	2.66 (2.35, 3.00)	**<0.001**	2.37 (2.11, 2.66)	**<0.001**
Physical Illness	3.88 (3.48, 4.34)	**<0.001**	4.17 (3.77, 4.63)	**<0.001**
Hallucinations	0.87 (0.74, 1.02)	0.08	0.82 (0.71, 0.96)	**0.01**
Depressed Mood	0.75 (0.67, 0.83)	**<0.001**	0.88 (0.80, 0.98)	**0.02**
Relationship Problems	0.65 (0.58, 0.74)	**<0.001**	0.71 (0.63, 0.79)	**<0.001**
Daily Living Problems	2.41 (2.16, 2.69)	**<0.001**	2.61 (2.36, 2.89)	**<0.001**
Living Conditions Problems	0.93 (0.80, 1.08)	0.34	0.89 (0.77, 1.02)	0.10
Occupational Problems	1.30 (1.15, 1.47)	**<0.001**	1.30 (1.16, 1.45)	**<0.001**
Overall increase in one unit of HoNoS	1.04 (1.03, 1.05)	**<0.001**	1.04 (1.04, 1.05)	**<0.001**
**Admitted to general hospital (within one year before or after Mood [affective] disorders diagnosis)**				

For peer review only

Ischaemia +CHD+ IHD	5.25 (4.54, 6.06)	**<0.001**	5.17 (4.53, 5.90)	**<0.001**
Arrhythmia + AF	6.30 (5.43, 7.31)	**<0.001**	6.10 (5.32, 6.99)	**<0.001**
Heart failure	7.81 (6.29, 9.68)	**<0.001**	7.89 (6.49, 9.59)	**<0.001**
Diabetes	4.66 (4.09, 5.31)	**<0.001**	4.18 (3.69, 4.73)	**<0.001**
Hypotension	7.29 (5.98, 8.90)	**<0.001**	6.51 (5.39, 7.86)	**<0.001**
Hypercholesterolemia	5.14 (4.47, 5.92)	**<0.001**	4.24 (3.70, 4.87)	**<0.001**
Hypertension	6.08 (5.50, 6.72)	**<0.001**	5.78 (5.26, 6.34)	**<0.001**
Urinary tract infections (UTI)	7.24 (6.42, 8.17)	**<0.001**	7.36 (6.59, 8.21)	**<0.001**
Osteoporosis	7.75 (6.44, 9.32)	**<0.001**	36.39 (32.35, 40.94)	**<0.001**
Visual Disturbance and Blindness	5.25 (3.99, 6.90)	**<0.001**	3.78 (2.84, 5.03)	**<0.001**
Hearing Loss	5.57 (4.09, 7.60)	**<0.001**	6.45 (4.95, 8.41)	**<0.001**
Syncope or Collapse	6.17 (5.54, 6.86)	**<0.001**	4.27 (3.84, 4.76)	**<0.001**
Parkinson's Disease	9.83 (7.31, 13.23)	**<0.001**	5.60 (3.97, 7.89)	**<0.001**
Falls before Mood [affective] disorders diagnosis	8.88 (7.8, 10.12)	**<0.001**	7.03 (6.18, 7.99)	**<0.001**
Fractures before Mood [affective] disorders diagnosis	5.31 (4.62, 6.10)	**<0.001**	10.67 (9.61, 11.85)	**<0.001**
Increase in one attendance to A&E following Mood [affective] disorders diagnosis	1.01 (1.01, 1.01)	**<0.001**	1.01 (1.01, 1.01)	**<0.001**

For peer review only

Table 3: Two models showing predictors of first fall hospital admission among Mood [affective] disorders. (used stepwise removal of factors that were not significant at 0.05 P value).

	Falls			
	Model 1 (n=20,938)		Model 2 (n=22,414)	
**Characteristics**	**HR (95% CI)**	**P value**	**HR (95% CI)**	**P value**
**Age at the time of Mood [affective] disorders diagnosis**				
18- 34	Ref.		Ref.	
35- 49	1.59 (1.23, 2.06)	**<0.001**	1.58 (1.24, 2.02)	**<0.001**
50- 64	3.11 (2.40, 4.01)	**<0.001**	2.98 (2.33, 3.81)	**<0.001**
65- 79	8.07 (6.31, 10.33)	**<0.001**	7.65 (6.07, 9.64)	**<0.001**
80 & over	12.40 (9.51, 16.17)	**<0.001**	11.80 (9.25, 15.05)	**<0.001**
Non-European ethnicity	0.43 (0.36, 0.51)	**<0.001**	0.45 (0.38, 0.52)	**<0.001**
Deprivation quintile (IMD 2015)				
Least deprived quintile	Ref.			
2nd least deprived quintile	0.99 (0.83, 1.19)	0.92		
3rd most deprived quintile	1.14 (0.95, 1.36)	0.17		
2nd most deprived quintile	1.15 (0.96, 1.38)	0.13		
Most deprived quintile	1.00 (0.84, 1.20)	0.85		
**Medication prescribed (within one year before or after diagnosis of Mood [affective] disorders)**				
Anticholinergics received	0.95 (0.74, 1.22)	0.68		
Antihypertensive received	0.98 (0.79, 1.21)	0.82		
Antidepressants received	0.91 (0.74, 1.11)	0.36		
Anxiolytics and Hypnotics received	1.00 (0.80, 1.24)	0.97		
Analgesics received	1.36 (1.10, 1.68)	**0.01**	1.39 (1.22, 1.58)	**<0.001**

For peer review only - http://bmjopen.bmj.com/site/about/guidelines.xhtml

Increase in one type of polypharmacy	1.02 (0.87, 1.19)	0.80		
**Presence of other psychiatric conditions (within one year before or after diagnosis of Mood [affective] disorders)**				
F00- F03 (Dementia)	1.05 (0.86, 1.27)	0.64		
F20- F29 (Schizophrenia spectrum disorder)	1.07 (0.85, 1.34)	0.57		
F30-F31 (Bipolar affective disorder)	0.87 (0.72, 1.06)	0.16		
F40- F48 (Neurotic, stress-related and somatoform disorders)	0.74 (0.60, 0.91)	**0.01**	0.75 (0.61, 0.91)	**0.01**
F50 (Eating Disorders)	0.86 (0.36, 2.09)	0.74		
**One unit increase in HoNoS**				
Self-Injury	1.10 (0.92, 1.32)	0.30		
Cognitive Problems	1.25 (1.06, 1.46)	**0.01**	1.28 (1.12, 1.46)	**<0.001**
Physical Illness	1.23 (1.07, 1.42)	**<0.001**	1.25 (1.10, 1.42)	**<0.001**
Depressed Mood	0.80 (0.70, 0.92)	**<0.001**	0.83 (0.74, 0.93)	**<0.001**
Relationship Problems	0.98 (0.84, 1.14)	0.77		
Daily Living Problems	1.01 (0.87, 1.17)	0.94		
Occupational Problems	0.90 (0.78, 1.03)	0.13		
**Admitted to general hospital (within one year before or after diagnosis of Mood [affective] disorders)**				
Ischaemia +CHD+ IHD	0.95 (0.78, 1.15)	0.59		
Arrhythmia + AF	0.88 (0.73, 1.07)	0.20		
Heart failure	1.37 (1.05, 1.78)	**0.02**	1.27 (1.00, 1.63)	**0.05**
Diabetes	1.37 (1.16, 1.63)	**<0.001**	1.36 (1.16, 1.59)	**<0.001**
Hypotension	1.38 (1.09, 1.75)	**0.01**	1.34 (1.07, 1.68)	**0.01**
Hypercholesterolemia	1.16 (0.96, 1.39)	0.13		
Hypertension	1.05 (0.88, 1.23)	0.63		
Urinary tract infections (UTI)	1.33 (1.13, 1.56)	**<0.001**	1.33 (1.14, 1.54)	**<0.001**

For peer review only - http://bmjopen.bmj.com/site/about/guidelines.xhtml

Osteoporosis	1.41 (1.08, 1.83)	**0.01**	1.37 (1.11, 1.70)	**<0.001**
Visual Disturbance and Blindness	1.22 (0.86, 1.71)	0.26		
Hearing Loss	0.91 (0.64, 1.30)	0.61		
Syncope or Collapse	1.94 (1.71, 2.26)	**<0.001**	2.05 (1.79, 2.36)	**<0.001**
Parkinson's Disease	1.25 (0.89, 1.77)	0.20		
Falls before Mood [affective] disorders diagnosis	1.81 (1.51, 2.18)	**<0.001**	1.81 (1.54, 2.13)	**<0.001**
Fractures before Mood [affective] disorders diagnosis	0.98 (0.78, 1.22)	0.86		
Increase in one attendance to A&E following Mood [affective] disorders * diagnosis	1.01 (1.01, 1.01)	**<0.001**	1.01 (1.01, 1.01)	**<0.001**

For peer review only - http://bmjopen.bmj.com/site/about/guidelines.xhtml

Table 4: Two models showing predictors of first fracture hospital admission among Mood [affective] disorders patients.

	Model 1 (n=20,936)		Model 2 (n=22,322)	
**Characteristics**	**HR (95% CI)**	**P value**	**HR (95% CI)**	**P value**
**Age at the time of Mood [affective] disorders diagnosis**				
18- 34	Ref.		Ref.	
35- 49	1.27 (1.02, 1.57)	**0.03**	1.27 (1.03, 1.55)	**0.02**
50- 64	2.31 (1.85, 2.88)	**<0.001**	2.28 (1.85, 2.81)	**<0.001**
65- 79	5.75 (4.65, 7.11)	**<0.001**	5.55 (4.54, 6.77)	**<0.001**
80 & over	7.46 (5.90, 9.45)	**<0.001**	7.37 (5.93, 9.16)	**<0.001**
Female gender	1.21 (1.08, 1.36)	**<0.001**	1.15 (1.03, 1.29)	**0.01**
Non-European ethnicity	0.59 (0.51, 0.68)	**<0.001**	0.59 (0.51, 0.67)	**<0.001**
Deprivation quintile (IMD 2015)				
Least deprived quintile	Ref.			
2nd least deprived quintile	1.07 (0.91, 1.28)	0.41		
3rd most deprived quintile	1.11 (0.94, 1.32)	0.23		
2nd most deprived quintile	1.17 (0.99, 1.39)	0.07		
Most deprived quintile	1.00 (0.85, 1.19)	0.96		
**Medication prescribed (within one year before or after diagnosis of Mood [affective] disorders)**				
Anticholinergics received	1.08 (0.85, 1.26)	0.76		
Antihypertensive received	0.76 (0.65, 0.91)	**<0.001**	0.80 (0.70, 0.92)	**<0.001**
Antidepressants received	1.08 (0.91, 1.28)	0.37		
Analgesics received	1.28 (1.08, 1.52)	**<0.001**	1.33 (1.17, 1.51)	**<0.001**
Increase in one type of polypharmacy	1.01 (0.93, 1.11)	0.75		

For peer review only

**Presence of other psychiatric conditions (within one year before or after diagnosis of Mood [affective] disorders)**				
F00- F03 (Dementia)	1.14 (0.96, 1.41)	0.12		
F20- F29 (Schizophrenia spectrum disorder)	1.11 (0.91, 1.38)	0.30		
F30- F31 (Bipolar affective disorder)	0.81 (0.67, 0.98)	**0.03**	0.80 (0.68, 0.95)	**0.01**
F40- F48 (Neurotic, stress-related and somatoform disorders)	0.82 (0.68, 0.99)	**0.04**	0.83 (0.70, 0.99)	**0.04**
**One unit increase in HoNoS**				
Cognitive Problems	1.02 (0.87, 1.19)	0.83		
Physical Illness	1.36 (1.19, 1.56)	**<0.001**	1.48 (1.31, 1.67)	**<0.001**
Hallucinations	0.91 (0.76, 1.10)	0.34		
Depressed Mood	0.83 (0.72, 0.94)	**0.01**	0.91 (0.82, 1.00)	**0.05**
Relationship Problems	0.89 (0.77, 1.02)	0.10		
Daily Living Problems	1.12 (0.98, 1.29)	0.11		
Occupational Problems	0.91 (0.79, 1.04)	0.15		
**Admitted to general hospital (within one year before or after diagnosis of Mood [affective] disorders)**				
Ischaemia +CHD+ IHD	1.01 (0.85, 1.22)	0.88		
Arrhythmia + AF	1.03 (0.86, 1.23)	0.78		
Heart failure	1.44 (1.12, 1.86)	**0.01**	1.43 (1.14, 1.80)	**<0.001**
Diabetes	1.22 (1.04, 1.44)	**0.02**	1.23 (1.06, 1.44)	**<0.001**
Hypotension	1.18 (0.93, 1.48)	0.17		
Hypercholesterolemia	0.85 (0.71, 1.03)	0.10		
Hypertension	1.05 (0.90, 1.23)	0.53		
Urinary tract infections (UTI)	1.49 (1.28, 1.74)	**<0.001**	1.52 (1.32, 1.75)	**<0.001**
Osteoporosis	7.31 (5.82, 9.18)	**<0.001**	7.00 (5.65, 8.70)	**<0.001**
Visual Disturbance and Blindness	1.06 (0.74, 1.53)	0.74		
Hearing Loss	1.40 (1.03, 1.92)	**0.03**	1.46 (1.09, 1.96)	**0.01**

For peer review only - http://bmjopen.bmj.com/site/about/guidelines.xhtml

Syncope or Collapse	1.18 (1.02, 1.36)	**0.03**		
Parkinson's Disease	0.85 (0.58, 1.25)	0.41		
Falls before Mood [affective] disorders diagnosis	1.02 (0.85, 1.22)	0.83		
Fractures before Mood [affective] disorders diagnosis	1.26 (1.01, 1.57)	**0.04**	1.33 (1.10, 1.63)	**<0.001**
Increase in one attendances to A&E following Mood [affective] disorders diagnosis	1.01 (1.01, 1.01)	**<0.001**	1.01 (1.01, 1.01)	**<0.001**

For peer review only - http://bmjopen.bmj.com/site/about/guidelines.xhtml

The RECORD statement – checklist of items, extended from the STROBE statement, that should be reported in observational studies using routinely collected health data.

	Item No.	STROBE items	Location in manuscript where items are reported	RECORD items	Location in manuscript where items are reported
**Title and abstract**					
	1	(a) Indicate the study's design with a commonly used term in the title or the abstract (b) Provide in the abstract an informative and balanced summary of what was done and what was found	Page 1 and 2	RECORD 1.1: The type of data used should be specified in the title or abstract. When possible, the name of the databases used should be included.<br><br>RECORD 1.2: If applicable, the geographic region and timeframe within which the study took place should be reported in the title or abstract.<br><br>RECORD 1.3: If linkage between databases was conducted for the study, this should be clearly stated in the title or abstract.	Page 2
**Introduction**					
Background rationale	2	Explain the scientific background and rationale for the investigation being reported	Page 4		
Objectives	3	State specific objectives, including any prespecified hypotheses	Page 4		
**Methods**					
Study Design	4	Present key elements of study design early in the paper	Page 5		
Setting	5	Describe the setting, locations, and relevant dates, including periods of recruitment, exposure, follow-up, and data collection	Page 5		
Participants	6	*(a) Cohort study* - Give the eligibility criteria, and the sources and methods of selection of participants. Describe [illegible]	Page 5	RECORD 6.1: The methods of study population selection (such as codes or algorithms used to identify subjects) should be listed in detail. If this is not [illegible]	Page 5

Variables	7	Clearly define all outcomes, exposures, predictors, potential confounders, and effect modifiers. Give diagnostic criteria, if applicable.	Page 6-8	RECORD 7.1: A complete list of codes and algorithms used to classify exposures, outcomes, confounders, and effect modifiers should be provided. If these cannot be reported, an explanation should be provided.	Page 6-8
Data sources/ measurement	8	For each variable of interest, give sources of data and details of methods of assessment (measurement).<br>Describe comparability of assessment methods if there is more than one group	Page 6-8		
Bias	9	Describe any efforts to address potential sources of bias	Page 9		
Study size	10	Explain how the study size was arrived at	Page 5		
Quantitative variables	11	Explain how quantitative variables were handled in the analyses. If applicable, describe which groupings were chosen, and why	Page 6-8		

For peer review only

Statistical methods	12	(a) Describe all statistical methods, including those used to control for confounding<br>(b) Describe any methods used to examine subgroups and interactions<br>(c) Explain how missing data were addressed<br>(d) *Cohort study* - If applicable, explain how loss to follow-up was addressed<br>*Case-control study* - If applicable, explain how matching of cases and controls was addressed<br>*Cross-sectional study* - If applicable, describe analytical methods taking account of sampling strategy<br>(e) Describe any sensitivity analyses	Page 9		
Data access and cleaning methods		..		RECORD 12.1: Authors should describe the extent to which the investigators had access to the database population used to create the study population.<br><br>RECORD 12.2: Authors should provide information on the data cleaning methods used in the study.	Page 5
Linkage		..		RECORD 12.3: State whether the study included person-level, institutional-level, or other data linkage across two or more databases. The methods of linkage and methods of linkage quality evaluation should be provided.	Page 6
**Results**					

For peer review only

Participants	13	(a) Report the numbers of individuals at each stage of the study (*e.g.*, numbers potentially eligible, examined for eligibility, confirmed eligible, included in the study, completing follow-up, and analysed)<br>(b) Give reasons for non-participation at each stage.<br>(c) Consider use of a flow diagram	Page 9	RECORD 13.1: Describe in detail the selection of the persons included in the study (*i.e.,* study population selection) including filtering based on data quality, data availability and linkage. The selection of included persons can be described in the text and/or by means of the study flow diagram.	Page 9
Descriptive data	14	(a) Give characteristics of study participants (*e.g.*, demographic, clinical, social) and information on exposures and potential confounders<br>(b) Indicate the number of participants with missing data for each variable of interest<br>(c) *Cohort study* - summarise follow-up time (*e.g.*, average and total amount)	Table 1 and page 9		
Outcome data	15	*Cohort study* - Report numbers of outcome events or summary measures over time<br>*Case-control study* - Report numbers in each exposure category, or summary measures of exposure<br>*Cross-sectional study* - Report numbers of outcome events or	Page 10		
		summary measures			

For peer review only

Main results	16	(a) Give unadjusted estimates and, if applicable, confounder-adjusted estimates and their precision (e.g., 95% confidence interval). Make clear which confounders were adjusted for and why they were included<br>(b) Report category boundaries when continuous variables were categorized<br>(c) If relevant, consider translating estimates of relative risk into absolute risk for a meaningful time period	Page 10-11		
Other analyses	17	Report other analyses done—e.g., analyses of subgroups and interactions, and sensitivity analyses	Not applicable		
**Discussion**					
Key results	18	Summarise key results with reference to study objectives	Page 13-16		
Limitations	19	Discuss limitations of the study, taking into account sources of potential bias or imprecision. Discuss both direction and magnitude of any potential bias	Page 16-17	RECORD 19.1: Discuss the implications of using data that were not created or collected to answer the specific research question(s). Include discussion of misclassification bias, unmeasured confounding, missing data, and changing eligibility over time, as they pertain to the study being reported.	Page 16-17
Interpretation	20	Give a cautious overall interpretation of results considering objectives, limitations, multiplicity of analyses, results from similar studies, and other relevant evidence	Page 17-18		
Generalisability	21	Discuss the generalisability (external validity) of the study results	Page 17		

For peer review only

Other Information					
Funding	22	Give the source of funding and the role of the funders for the present study and, if applicable, for the original study on which the present article is based	Page 18		
Accessibility of protocol, raw data, and programming code		..		RECORD 22.1: Authors should provide information on how to access any supplemental information such as the study protocol, raw data, or programming code.	Page 19

*Reference: Benchimol EI, Smeeth L, Guttmann A, Harron K, Moher D, Petersen I, Sørensen HT, von Elm E, Langan SM, the RECORD Working Committee. The REporting of studies Conducted using Observational Routinely-collected health Data (RECORD) Statement. *PLoS Medicine* 2015; in press.

*Checklist is protected under Creative Commons Attribution (CC BY) license.

For peer review only

Supplementary material Table 1. Mean age at the time of fall/ fracture and mood disorder diagnosis among patients with Mood [affective] disorders

	Presence of falls		Presence of fractures	
Age	**No (n= 34,270)**	**Yes (n= 1,831)**	**No (n= 33,908)**	**Yes (n= 2,193)**
Mean age at diagnosis (SD)	43.4 (17.1)	62.4 (19.9)	43.3 (17.1)	60.6 (20.6)
Mean age at fall/ fracture/ (SD)		64.2 (20.8)		62.5 (22.8)

For peer review only
